# Sesquiterpenes with new carbon skeletons from the basidiomycete *Phlebia tremellosa*

**DOI:** 10.1007/s11418-019-01286-8

**Published:** 2019-02-19

**Authors:** Ken-ichi Nakashima, Junko Tomida, Takao Hirai, Yoshiaki Kawamura, Makoto Inoue

**Affiliations:** 1 Laboratory of Medicinal Resources, School of Pharmacy, Aichi Gakuin University, 1-100 Kusumoto-cho, Chikusa-ku, Nagoya, Aichi 464-8650 Japan; 2 Department of Microbiology, School of Pharmacy, Aichi Gakuin University, 1-100 Kusumoto-cho, Chikusa-ku, Nagoya, Aichi 464-8650 Japan

**Keywords:** Basidiomycetes, *Phlebia tremellosa*, Sesquiterpenoids, Seco-sterpurane, Phlebiane

## Abstract

Three new sesquiterpenes, phlebidiol, phlebioic acid, and phlebiolide, as well as the known compound tremetriol, were isolated from cultures of the basidiomycete *Phlebia tremellosa*. The structures of all isolated compounds were established by extensive spectroscopic analyses, including those involving extensive two-dimensional nuclear magnetic resonance. The absolute configurations of phlebidiol, phlebioic acid, and phlebiolide were determined by comparisons of experimental and calculated electronic circular dichroism spectra. Phlebidiol and phlebioic acid have previously unreported carbon skeletons, for which we propose the skeletal names “seco-sterpurane” and “phlebiane,” respectively. Phlebiolide is also the second published example of a merulane sesquiterpene.

**Electronic supplementary material:**

The online version of this article (10.1007/s11418-019-01286-8) contains supplementary material, which is available to authorized users.

## Introduction

Basidiomycetes is a major class of fungi, together with Ascomycetes, and is a rich source of unique secondary metabolites. *Phlebia tremellosa* (Schrad.) Nakasone & Burds. (formerly *Merulius tremellosus*), a species in class Basidiomycetes, is classified in the family Meruliaceae and is also known to be one of the wood-decay fungi [1, 2]. Sesquiterpenes with rare skeletons, such as sterpurane, isolactarane, and merulane sesquiterpenes, have been identified in previous studies to be secondary products of *P. tremellosa* or *P. uda* [3–5]. Although thousands of sesquiterpenes have been identified in the literature, sterpurane and isolactarane sesquiterpenes are rarely found in nature [6]. Following the isolation of the first sterpurane, sterpuric acid, from *Chondrostereum purpureum* (formerly *Stereum purpureum*) in 1981 [7], sterpurane sesquiterpenes have been isolated from the basidiomycetes *Artomyces pyxidatus* (formerly *Clavicorona pyxidata*) [8], *Flammulina velutipesin* [9], and *Gloeophyllum* sp. [10], as well as from *Phlebia* spp. and the soft coral *Alcyonium acaule* [11]. Isolactarorufin, the first isolactarane to be identified, was isolated from *Lactarius rufus* in 1976 [12]. Isolactarane sesquiterpenes have since been isolated from the basidiomycetes *Flammulina velutipesin* [8] and *Phlebia* spp., as have sterpurane sesquiterpenes, and have also been detected in *Hyphodontia* sp. [13], *Russula delica* [14], and other *Lactarius* spp. [15]. The only known sesquiterpene with a merulane skeleton is meruliolactone, which was isolated from cultures of *Phlebia tremellosa* [3].

**Fig. 1:**
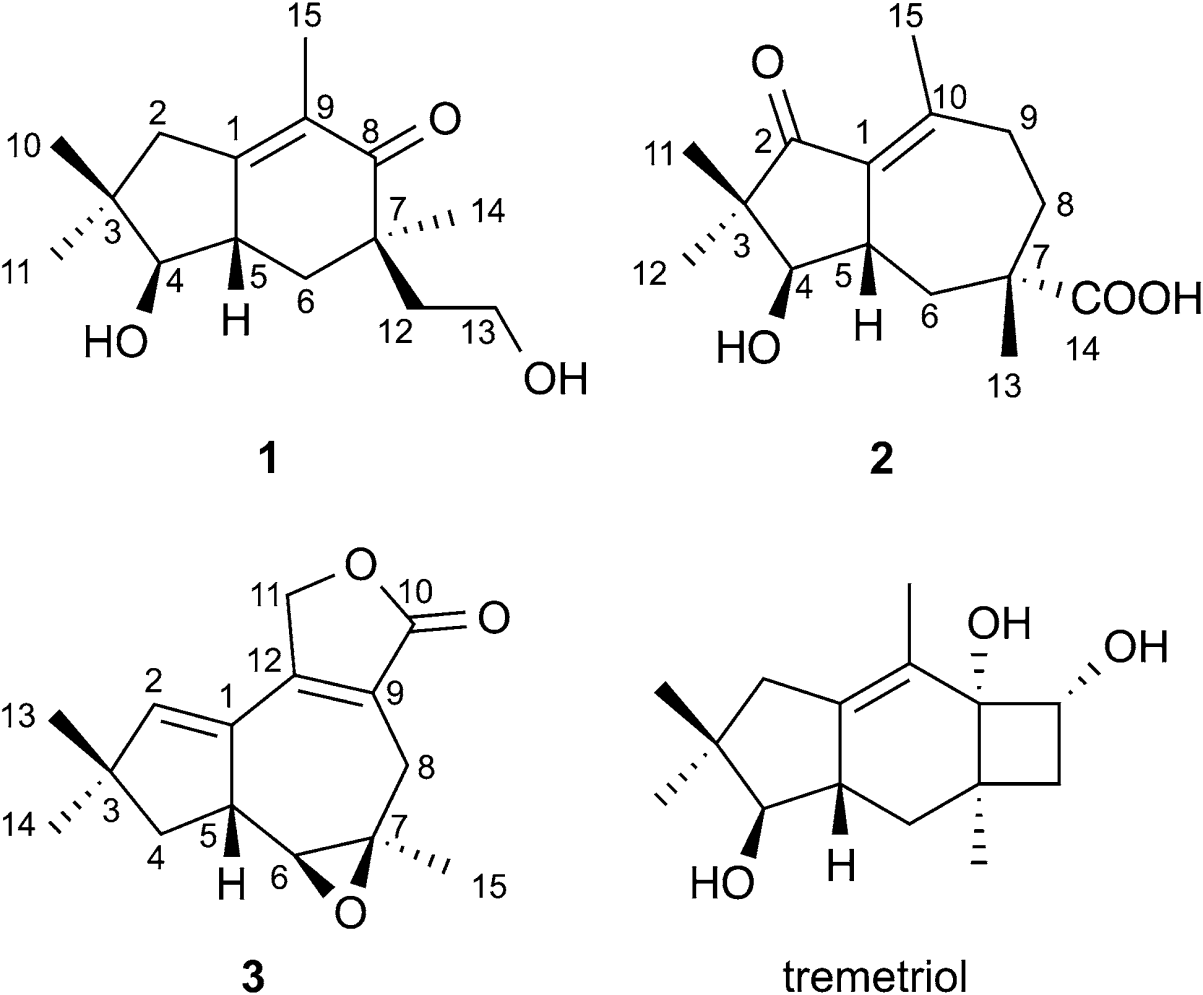
Chemical structures of compounds isolated from *Phlebia tremellosa*

As part of our research on the secondary metabolites of plant-associated endophytic fungi [16–19], we isolated and cultured the basidiomycete *Phlebia tremellosa* from the leaves of *Senna alata* (Fabaceae) and succeeded in isolating three new sesquiterpenes, namely phlebidiol (**1**), phlebioic acid (**2**), and phlebiolide (**3**), along with a known sterpurane sesquiterpene from solid cultures of *P. tremellosa* ECN184 (Fig. 1). Compounds **1** and **2** have unprecedented carbon skeletons, for which we propose the skeletal names “seco-sterpurane” and “phlebiane”, respectively. Furthermore, **3** is the second published example of a merulane sesquiterpene.

## Results and discussion

*Phlebia tremellosa* ECN184 was isolated from the healthy leaves of *Senna alata* and identified by sequencing the D1/D2 26S rRNA gene and internal transcript spacers (ITS) of the ribosomal DNA. The whole mycelia of *P. tremellosa*, which were cultured on 180 plates of 2% malt extract agar (MEA) for 30 days, were extracted three times with MeOH at room temperature and concentrated under reduced pressure to afford the crude extract. The MeOH extract was then partitioned between ethyl acetate and water. The ethyl acetate extract was subjected to repeated silica gel and Sephadex LH-20 column chromatography (CC) to obtain three new compounds (**1**–**3**). In addition, the known compound tremetriol was also isolated and identified based on comparisons of spectroscopic data with spectra published in the literature [3].

**Fig. 2:**
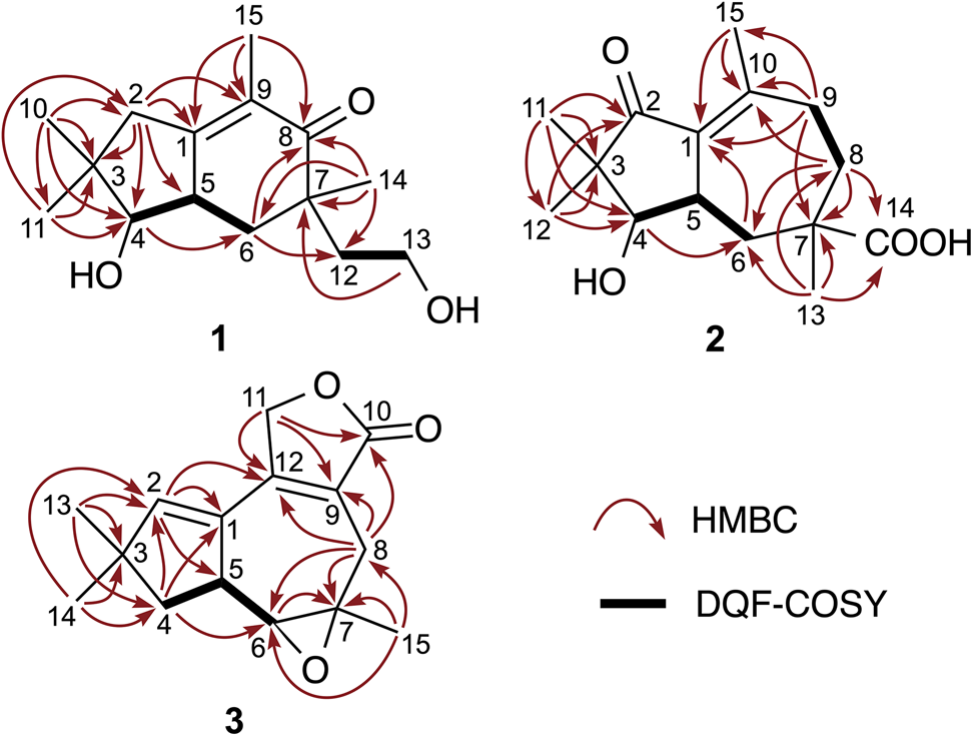
Key HMBC (arrows) and double-quantum filtered (DQF)–COSY (bold) correlations in **1**–**3**

**Fig. 3:**
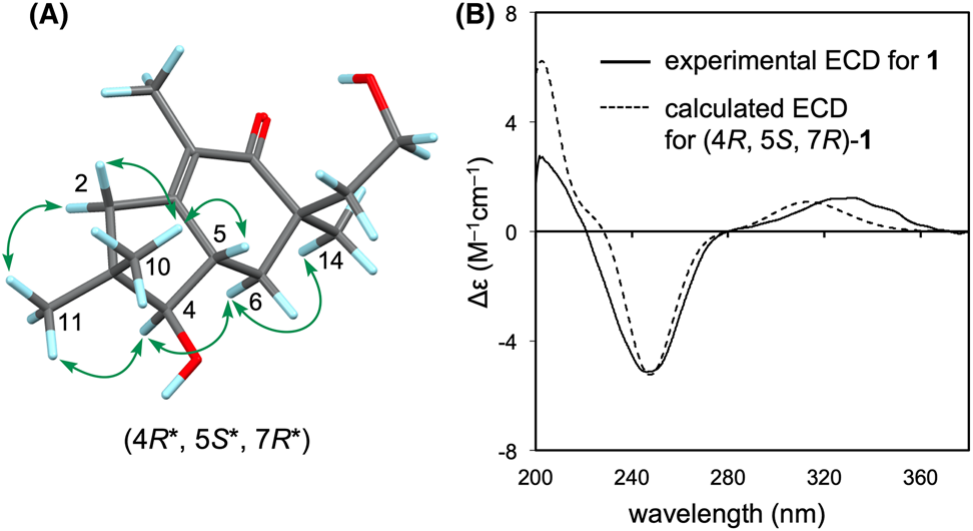
**a** Key NOESY correlations (arrows) in phlebidiol (**1**). **b** Comparing the experimental electronic circular dichroism (ECD) spectrum of **1** with the calculated ECD spectrum of (4*R*, 5*S*, 7*R*)-**1**

Phlebidiol (**1**), ${[\alpha]{^{20}_{D}}}$ = + 8.4, was isolated as a colorless oil. Its molecular formula (C_15_H_24_O_3_) was established from high-resolution electrospray ionisation mass spectrometry (HRESIMS) data (*m*/*z* 275.1607, [M+Na]^+^, calcd 275.1623). The IR spectrum showed absorptions indicating the presence of hydroxy groups (3402 cm^−1^) and a carbonyl group (1645 cm^−1^). The ^13^C NMR and distortionless enhancement by polarization transfer (DEPT)-135 spectra showed the presence of four methyls, four methylenes, two methines, and five nonprotonated carbons, including three *sp*^2^ carbons of a tetra-substituted double bond at *δ*_C_ 159.9 (C-1) and 128.4 (C-9), as well as a ketonic carbon at *δ*_C_ 204.1 (C-8). All protonated carbons were assigned on the basis of the HMQC spectra (Table 1). According to the four degrees of unsaturation and the substructures indicated by the NMR spectra, **1** was proposed to be a bicyclic sesquiterpene. The ^1^H-NMR data combined with the double-quantum filtered (DQF)–COSY spectrum suggested C-4/C-5/C-6 and C-12/C-13 carbon-chain sequences (Fig. 2). HMBC correlations from one methyl proton signal [*δ*_H_ 1.64 (3H, br s, H_3_–15)] to C-1, C-8, and C-9 demonstrated the presence of an α-methyl enone moiety, while another methyl resonance [*δ*_H_ 1.16 (3H, s, H_3_–14)] exhibited four HMBC correlations to two methylene carbons at *δ*_C_ 40.7 (C-6) and 37.8 (C-12), a ketonic carbon (C-8), as well as an undistinguishable carbon signal assigned to a methine carbon at *δ*_C_ 42.8 (C-5) or a quaternary carbon at *δ*_C_ 42.6 (C-7). The observation of four correlations from a methyl-proton singlet indicated a linkage between C-14 and a quaternary carbon, i.e., C-7. Accordingly, the remaining carbon atoms C-6, C-8, and C-12 that correlated with H_3_-14 were all assumed to be bound to C-7. HMBC correlations from H_2_-6 [*δ*_H_ 2.19 (1H, dd, *J* = 4.8, 13.0 Hz); 1.55 (1H, dd, *J* = 13.0, 14.8 Hz)] to C-8, C-12, and C-14 and from H_2_-13 [*δ*_H_ 3.73 (2H, m)] to C-7 also sustained the partial structure around C-7. The other methyl proton signals [*δ*_H_ 1.02 (3H, s, H_3_-10) and *δ*_H_ 1.14 (3H, s, H_3_-11)] exhibited HMBC correlations with C-2, C-3, C-4, and C-11/C-10. In addition, H_2_-2 [*δ*_H_ 2.46 and 2.25 (1H each, d, *J* = 19.4 Hz)] correlated with C-1, C-3, C-4, C-5, and C-9, which was suggestive of linkages from C-1 to C-4 and between C-1 and C-5, as well as the presence of two methyl groups at C-3. Hence, the structure of phlebidiol (**1**) was proposed to be a four-membered ring-opening product of a sterpurane sesquiterpene. The relative configuration of **1** was confirmed by the essential NOEs [H-2β/H_3_-10; H_3_-10/H-5; H-2α/H_3_-11; H_3_-11/H-4; H-4/H-6α; H-6α/H_3_-14] observed in the NOESY spectrum (Fig. 3a).

**Table 1:** ^1^H and ^13^C NMR data (400 and 100 MHz) of compounds **1**–**3** in deuterochloroform (CDCl_3_)

No.	Phlebidiol (**1**)		Phlebioic acid (**2**)		Phlebiolide (**3**)	
	*δ*_H_ (*J* in Hz)	*δ*_C_	*δ*_H_ (*J* in Hz)	*δ*_C_	*δ*_H_ (*J* in Hz)	*δ*_C_
1		159.9		132.8		132.0
2α	2.25 (1H, d, *J* = 19.4)	43.4		208.2	5.84 (1H, d, *J* = 2.0)	144.9
2β	2.46 (1H, d, *J* = 19.4)					
3		39.5		49.4		46.3
4α	3.35 (1H, d, *J* = 10.4)	84.4	3.43 (1H, d, *J* = 9.2)	81.2	2.33 (1H, dd, *J* = 8.8, 13.6)	45.1
4β					1.75 (1H, dd, *J* = 6.8, 13.6)	
5	2.86 (1H, m)^a^	42.8	2.70 (1H, m)^b^	41.2	3.21 (1H, m)	47.9
6α	1.55 (1H, dd, *J* = 13.0, 14.8)	40.7	2.08 (2H, m)	38.9	2.66 (1H, d, *J* = 6.8)	66.0
6β	2.19 (1H, dd, *J* = 4.8, 13.0)					
7		42.6		44.2		61.0
8α		204.1	2.10 (1H, m)	34.0	2.95 (1H, d, *J* = 15.6)	30.5
8β			1.77 (1H, br t, *J* = 12.8)		2.57 (1H, br d, *J* = 15.6)^c, d^	
9α		128.4	2.54 (1H, dd, *J* = 12.4, 18.0)	34.7		122.9
9β			2.28 (1H, dd, *J* = 6.8, 18.0)			
10	1.02 (3H, s)	21.9		155.8		174.3
11	1.14 (3H, s)	27.4	1.00 (3H, s)	17.8	4.82 (1H, dd, *J* = 2.4, 16.1)^c^	69.2
					4.87 (1H, dd, *J* = 2.4, 16.1)^d^	
12	1.85 (1H, dt, *J* = 6.8, 13.6)	37.8	1.11 (3H, s)	21.7		153.8
	1.64 (1H, m)					
13	3.73 (2H, m)	59.2	1.35 (3H, s)	24.8	1.23 (3H, s)	29.6
14	1.16 (3H, s)	23.6		182.8	1.11 (3H, s)	28.1
15	1.64 (3H, br s)^a^	11.2	2.19 (3H, d, *J* = 1.6)^b^	22.3	1.33 (3H, s)	23.0

^a–d^Signals are coupled homoallylically with each other

To elucidate the absolute configuration of **1**, the electronic circular dichroism (ECD) spectrum was calculated by quantum-chemical methods and compared with the experimental spectrum. The preliminary conformers, which were generated using the GMMX add-on module (energy window = 10 kcal/mol), were optimized using density functional theory (DFT) at the CAM-B3LYP/6-31+G(d,p) level. ECD spectra of the DFT-optimized conformers were calculated using time-dependent DFT at the CAM-B3LYP/6-31+G(d,p) level with the conductor-like polarizable continuum model (CPCM) solvation model in MeOH. The obtained ECD spectra were Boltzmann averaged on the basis of the calculated relative energies of the DFT-optimized conformers. The experimental and calculated ECD spectra of **1** were in good agreement for the (4*R*, 5*S*, 7*R*) absolute configuration (Fig. 3b).

**Fig. 4:**
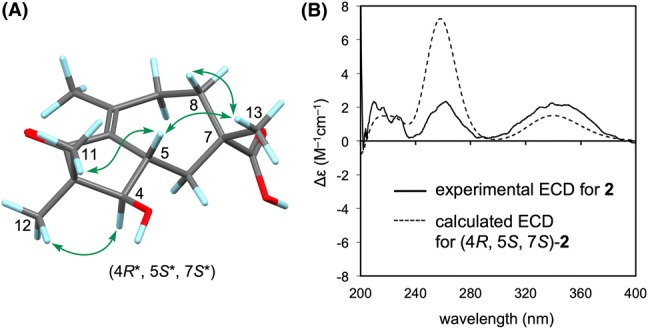
**a** Key NOESY correlations (arrows) in phlebioic acid (**2**). **b** Comparison of the experimental ECD spectrum of **2** with the calculated ECD spectrum of (4*R*, 5*S*, 7*S*)-**2**

Phlebioic acid (**2**), ${[\alpha]{^{20}_{D}}}$ = + 173.8, was obtained as a colorless oil. HRESIMS revealed an [M+Na]^+^ ion peak at 289.1396, indicating the quasi-molecular formula C_15_H_22_O_4_Na (calcd 289.1416). The IR spectrum exhibited absorptions for hydroxy groups (3435 cm^−1^) and carbonyl groups (1699 cm^−1^). The ^13^C NMR and DEPT135 spectra (Table 1) showed the presence of four methyls, three methylenes, two methines, and six nonprotonated carbons, including four* sp*^2^ carbons assigned to a tetra-substituted double bond [*δ*_C_ 132.8 (C-1) and 155.8 (C-10)], a carboxy group [*δ*_C_ 182.8 (C-14)], and a ketonic carbonyl group [*δ*_C_ 208.2 (C-2)]. The DQF–COSY spectrum showed two solitary C-4/C-5/C-6 and C-8/C-9 sequences (Fig. 2). HMBC correlations [H_2_-8/C-10; H_2_-9/C-1, C-10, C-15; H_3_-15/C-1, C-10] indicated that both C-9 and C-15 were bound to an *sp*^2^ carbon (C-10) within the double bond. HMBC correlations from H_3_-13 [*δ*_H_ 1.35 (3H, s)] to two methylene carbons (C-6 and C-8), a quaternary *sp*^3^ carbon (C-7), and a carboxy carbon (C-14) suggested the C-6/C-7/C-8 sequence, with both a methyl group and a carboxy group bonded to C-7, as sustained by the HMBC correlations [H_2_-8/C-6, C-7, C-14 and H_2_-9/C-7]. Furthermore, both methyl-proton signals [*δ*_H_ 1.00 (3H, s, H_3_-11) and *δ*_H_ 1.11 (3H, s, H_3_-12)] correlated with a ketonic carbon (C-2), a quaternary *sp*^3^ carbon (C-3), and a methylene carbon (C-4), suggestive of the C-2/C-3/C-4 sequence with two methyl groups at C-3. The HMBC correlation from H_2_-6 to C-1 confirmed the linkage between C-1 and C-5. The remaining indices of hydrogen deficiency and the chemical shifts of C-1 and C-10 implied a linkage between C-1 and C-2. Hence, the planar structure of **2** was determined to be a sesquiterpene with a previously unreported skeleton. The relative structure of **2** depicted in Fig. 4a was elucidated by the NOEs [H_3_-11/H-5; H-5/H_3_-13; H_3_-12/H-4]. Finally, we determined the absolute structure of **2** to be the (4*R*, 5*S*, 7*S*) configuration because the experimental ECD spectrum was in good agreement with the time-dependent density functional theory (TDDFT) spectrum calculated at the CAM-B3LYP/6-31+G(d,p)//CAM-B3LYP/6-311+G(d,p) level (Fig. 4b).

**Fig. 5:**
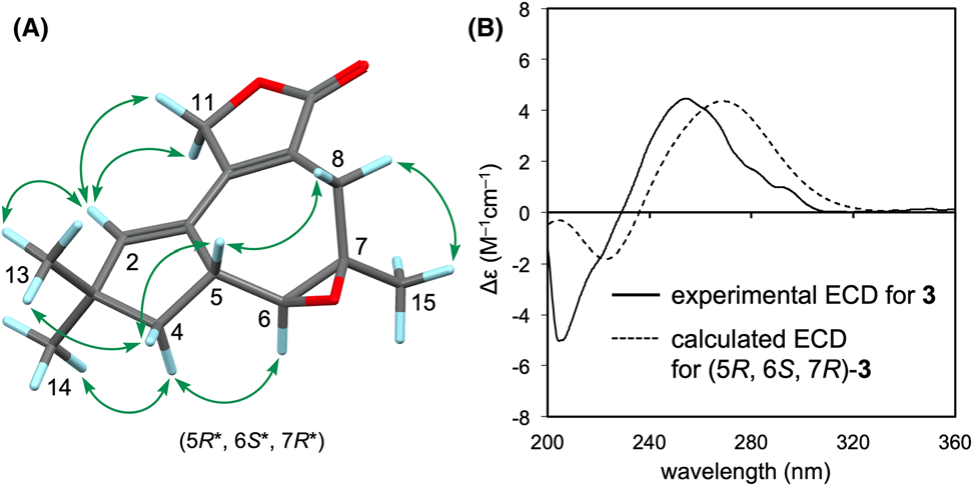
**a** Key NOESY correlations (arrows) in phlebiolide (**3**). **b** Comparison of the experimental ECD spectrum of **3** with the calculated ECD spectrum of (5*R*, 6*S*, 7*R*)-**3**

Phlebiolide (**3**), ${[\alpha]{^{20}_{D}}}$ = + 21.6, was obtained as a colorless oil. HRESIMS revealed an [M+Na]^+^ ion at *m*/*z* 269.1133, indicating the molecular formula C_15_H_18_O_3_Na (calcd 269.1154). The IR spectrum exhibited a strong absorption for a carbonyl group (1749 cm^−1^). The ^1^H NMR spectrum (Table 1) indicated the presence of three methyls, three methylenes, two *sp*^3^ methines, and an *sp*^2^ methine. Additionally, the ^13^C NMR spectrum displayed signals for 15 carbon atoms, which could be classified as three methyls, three methylenes, two *sp*^3^ and one *sp*^2^ methines, and two *sp*^3^ and four *sp*^2^ nonprotonated carbons, including a carbonyl carbon. The DQF–COSY spectrum revealed correlations indicative of the C-4/C-5/C-6 connection (Fig. 2). The HMBC spectrum showed correlations from both H_3_-13 and H_3_-14 to an olefinic methine (C-2), a quaternary carbon (C-3), and a methylene (C-4), as well as mutual correlations (C-14 or C-13). The HMBC correlations [H-2/C-1, C-5; H_2_-4/C-1, C-2] suggested a linkage between C-1 and C-5, revealing the presence of a 3,3-dimethylcyclopentene moiety. The HMBC spectrum also revealed correlations from the uncoupled methyl group [*δ*_H_ 1.33 (3H, s, H_3_-15)] to a methine at *δ*_C_ 66.0 (C-6), a nonprotonated carbon at *δ*_C_ 61.0 (C-7), and a methylene at *δ*_C_ 30.5 (C-8). Furthermore, H_2_-8 correlated with two *sp*^2^ quaternary carbons at *δ*_C_ 122.9 (C-9) and 153.8 (C-12) and a carbonyl carbon at *δ*_C_ 174.3 (C-10). The carbon signal of C-12 also correlated with an olefinic proton (H-2) within a 3,3-dimethylcyclopentene moiety. These correlations allowed a seven-membered ring bearing a methyl group and a carbonyl group at C-7 and C-9, respectively, to be constructed. The remaining oxygenated methylene (H_2_-11) was proposed to be attached to C-12 based on HMBC correlations from H_2_-11 to C-9 and C-12. The location of C-2 was also corroborated by an NOE between H-2 and H_2_-11, as observed in the NOESY spectrum. Finally, the correlation from H_2_-11 to C-10 confirmed the formation of a γ-lactone ring. The oxygen functional groups at C-6 and C-7 were determined to be an epoxy group based on the remaining indices of hydrogen deficiency and the chemical shifts in the ^1^H and ^13^C NMR spectra. The presence of the epoxy group was also supported by the fact that the IR spectrum showed no absorption for hydroxy groups. The relative configurations of C-5, C-6, and C-7 were deduced to be as shown in Fig. 5a by key NOEs [H_3_-13/H-4β; H-4β/H-5; H-5/H-8β; H_3_-14/H-4α; H-4α/H-6; H_3_-15/H-8α] in the NOESY spectrum. Based on the comparison of the experimental spectrum and the TDDFT ECD spectrum calculated at the CAM-B3LYP/6-31+G(d,p)//APFD/6-311+G(d,p) level of theory, the absolute structure of **3** was determined to have a (5*R*, 6*S*, 7*R*) configuration (Fig. 5b).

All isolated compounds exhibited no agonistic activities for peroxisome proliferator-activated receptor γ, retinoid X receptor α, and liver X receptor α in a luciferase reportor assay based on the method described in our previous paper [20]. Further studies of the biological activities of isolated compounds are in progress.

**Fig. 6:**
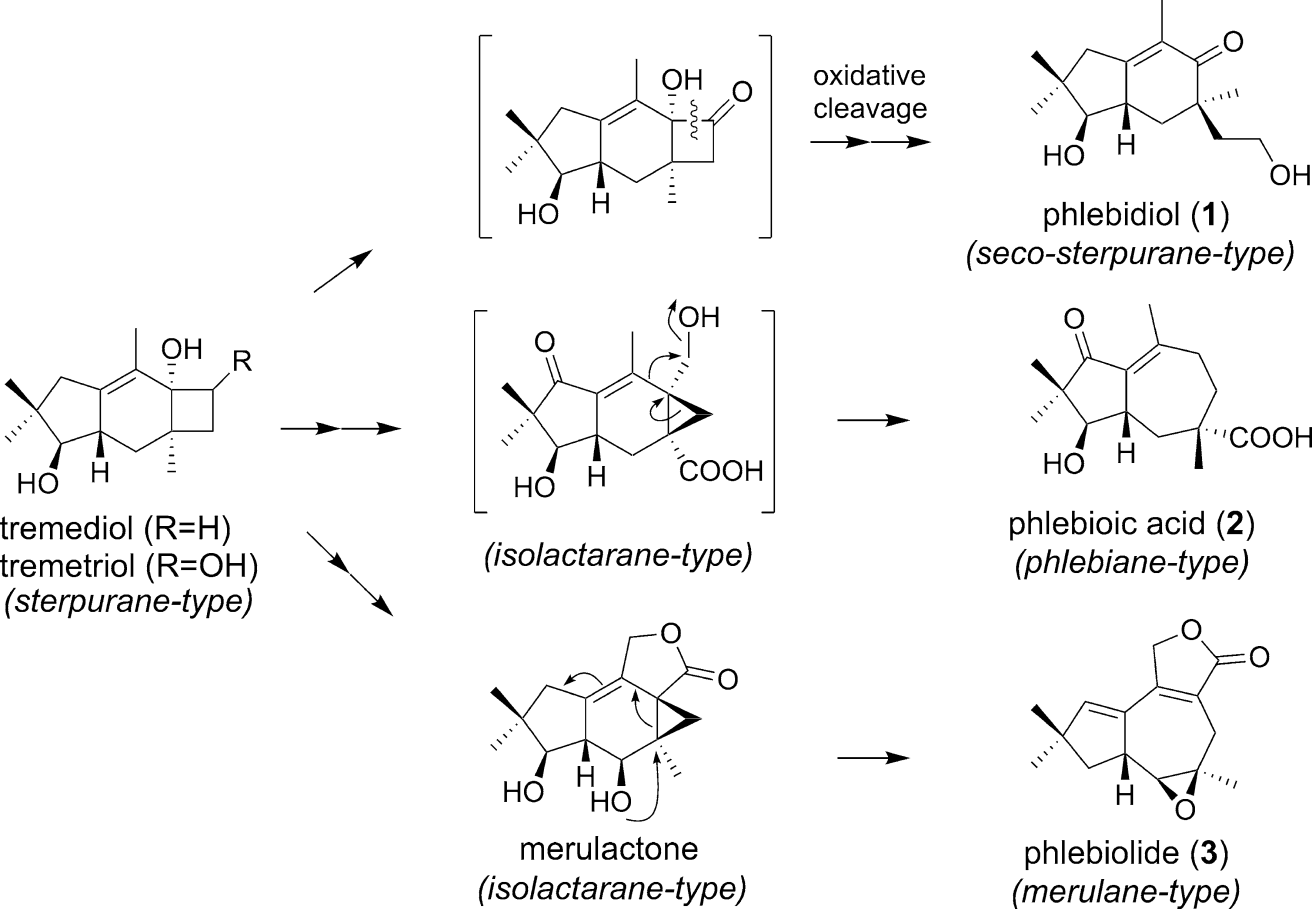
Plausible biogenetic pathways of **1**–**3**

In this study, we isolated three new sesquiterpenes with distinct carbon frameworks. Most notably, **1** and **2** have new skeletons that are named “seco-sterpurane” and “phlebiane”, respectively. The biogenetic pathway of phlebidiol (**1**), the first described seco-sterpurane, is possibly explained by the oxidative aliphatic C–C bond cleavage of the α-hydroxy ketones within the cyclobutane ring of an oxidized tremetriol (Fig. 6). A similar oxidative cleavage of a C–C bond was previously proposed in the clavicoronane sesquiterpenes [7]. Cytochrome P450 17A1 (CYP17A1) is an example of an enzyme that can cleave α-hydroxy ketones devoid of α-C–H bonds. CYP17A1 is a heme enzyme that catalyzes the cleavage of the C-17 to C-20 bond in 17α-hydroxypregnenolone during the biosynthesis of dehydroepiandrosterone via the ferric peroxo-hemiacetal intermediate [21, 22]. Metalloenzymes similar to CYP17A1 seem to be involved even in the biosynthesis of **1**. Meanwhile, the first phlebiane sesquiterpene, phlebioic acid (**2**), is potentially biosynthesized by the secondary ring-opening rearrangement of the isolactarane sesquiterpene, as shown in Fig. 6, although no plausible precursor has been isolated from *P. tremellosa*. Furthermore, the biosynthetic pathway to phlebiolide (**3**) also seems to involve the secondary ring-opening rearrangement of an isolactarane sesquiterpene. Merulactone [3], a previously isolated isolactarane from *P. tremellosa*, is likely to be the precursor of **3**; this biogenesis can be rationalized by rearrangement with ring-expansion initiated by the nucleophilic substitution of a hydroxyl group at the C-6 of merulactone. To the best of our knowledge, this is the first example of a proposed scaffold transformation from isolactarane to merulane involving a plausible precursor.

## Conclusion

Three novel sesquiterpenes (**1**–**3**) were isolated from the cultures of *Phlebia tremellosa*. The absolute configurations of **1**–**3** were confirmed by CD spectroscopy and DFT calculations. All new compounds are likely to have originated from sterpurane and/or isolactarane sesquiterpenes. Despite the fact that sterpurane and isolactarane sesquiterpenes have been isolated from several fungal species as mentioned previously, with the exception of merulactone, seco-sterpurane, phlebiane, and merulane sesquiterpenes have never been detected previously [23]. Therefore, our present study revealed the existence of distinctive biosynthetic pathways for the production of rare sesquiterpenes in the basidiomycete *P. tremellosa*.

## Experimental

### General experimental procedures

Optical rotation values were recorded on a P-1020 polarimeter (JASCO Corp., Yokyo, Japan). UV spectra were obtained with a Hitachi U-2900 spectrometer (Hitachi Ltd., Tokyo, Japan). ECD spectra were acquired on a JASCO J-820 spectropolarimeter (JASCO Corp.). IR spectra were recorded on a FTIR-8400S spectrophotometer (Shimadzu Ltd., Kyoto, Japan). NMR spectra were acquired on models JNM-AL-400 and JNM-ECZ 400S spectrometers with tetramethylsilane as the internal standard (JEOL Ltd., Tokyo, Japan). Electrospray ionization-MS data were obtained with a LCMS-IT-TOF mass spectrometer (Shimadzu Ltd.). DNA sequencing was performed with a model 3130 genetic analyzer (Applied Biosystems, Foster City, CA, USA). Silica gel AP-300 (Toyota Kako, Tokyo, Japan) and Sephadex LH-20 (GE Healthcare, Chicago, IL, USA) resins were used for CC. Silica gel 60 F_254_ and RP-18 F_254S_ (Merck & Co., Inc., Kenilworth, NJ, USA) were used for TLC.

### Fungal material and identification

*Phlebia tremellosa* ECN184 was isolated from the healthy leaves of *Senna alata* cultivated in the Herbal Garden of Gifu Pharmaceutical University (Gifu, Japan) in November 2016. The surfaces of the leaves were sterilized by sequential soaking in 95% EtOH for 30 s, 0.5% NaClO for 2 min, and 70% EtOH for 2 min. The surface-sterilized leaves were cut into 1-cm^2^ pieces and cultured on MEA containing 2% malt extract, 0.1% bacto peptone, 2% d-glucose, and 1.5% agar supplemented with 0.005% chloramphenicol in 9-cm petri dishes. The dishes were then incubated at 27 °C. Emergent organisms were isolated on new MEA. On the basis of the DNA sequencing of the ITS of rDNA and the D1/D2 domain of the 26S rDNA, the isolate belonged to genus* Phlebia*. The sequence data of *P. tremellosa* have been deposited at the DNA Data Bank of Japan (DDBJ) under the access numbers LC424440 (26S rRNA) and LC424443 (ITS). The strain was deposited at Department of Microbiology, School of Pharmacy, Aichi Gakuin University (ECN-184).

### Fermentation, extraction, and isolation

The fungus *P. tremellosa* was inoculated onto 150 MEA plates without chloramphenicol. After incubation at 27 °C for 30 days, the fermented materials were extracted with MeOH (2 × 4 L, each 24 h) at room temperature, and the solution was evaporated in vacuo to obtain the MeOH extract (71.8 g). The MeOH extract was partitioned three times with equal amounts of ethyl acetate and water, and the ethyl acetate solution was concentrated under vacuum to yield the ethyl acetate soluble fraction (7.2 g). The ethyl acetate fraction was separated on a silica gel column with CHCl_3_/MeOH (gradient 50:1 to 8:1, v/v) as the eluent, to give fractions (Frs.) 1–12. Fr. 8 was purified with silica gel CC (*n*-hexane/ethyl acetate 5:1 to 3:1, v/v) to obtain phlebiolide (**3**; 2.1 mg). Fr. 9 was subjected to silica gel column purification (*n*-hexane/ethyl acetate 5:1 to 3:1, v/v) to obtain tremetriol (4.5 mg). Phlebioic acid (**2**; 2.1 mg) was isolated from Fr. 10 with the aid of a silica gel column (*n*-hexane/acetone, 1:1). Fr. 11 was purified with a Sephadex LH-20 column (MeOH) to yield phlebidiol (**1**; 41.4 mg).

#### *Phlebidiol *(**1**)

Colorless oil; ${[\alpha]{^{20}_{D}}}$+ 8.4 (*c* 0.1, MeOH); UV (MeOH) *λ*_max_ (log *ε*) 246 (3.73), 290sh (2.56); IR (KBr) *ν*_max_ 3402, 2957, 2932, 2870, 1645, 1459, 1380, 1062, 1041 cm^−1^; ECD (0.02 mg/mL, MeOH) *λ*_ext_ (Δ*ε*) 328 (+ 1.20), 247 (–5.24), 203 (+ 2.68) nm; ^1^H NMR (400 MHz) and ^13^C NMR (100 MHz) data, see Table 1; HRESIMS *m*/*z* 275.1607 [M+Na]^+^ (calcd for C_15_H_24_O_3_Na, 275.1623).

#### *Phlebioic acid* (**2**)

Colorless oil;${[\alpha]{^{20}_{D}}}$ + 173.8 (*c* 0.1, MeOH); UV (MeOH) *λ*_max_ (log *ε*) 257 (4.03) nm; IR (KBr) *ν*_max_ 3435, 2969, 2932, 2874, 1699, 1616, 1464, 1211, 1078 cm^−1^; ECD (0.02 mg/mL, MeOH) *λ*_ext_ (Δ*ε*) 346 (+ 2.01), 261 (+ 2.31), 227 (+ 1.42), 217 (+ 1.96), 210 (+ 2.31) nm; ^1^H NMR (400 MHz) and ^13^C NMR (100 MHz) data, see Table 1; HRESIMS *m*/*z* 289.1396 [M+Na]^+^ (calcd for C_15_H_22_O_4_Na, 289.1416).

#### *Phlebiolide* (**3**)

Colorless oil; ${[\alpha]{^{20}_{D}}}$ + 21.6 (*c* 0.1, MeOH); UV (MeOH) *λ*_max_ (log *ε*) 275 (4.03) nm; IR (KBr) *ν*_max_ 2957, 2926, 2864, 1749, 1651, 1647, 1456, 1224, 1159, 1041 cm^−1^; ECD (0.02 mg/mL, MeOH) *λ*_ext_ (Δ*ε*) 254 (+ 4.47), 205 (– 5.01) nm; ^1^H NMR (400 MHz) and ^13^C NMR (100 MHz) data, see Table 1; HRESIMS *m*/*z* 269.1133 [M+Na]^+^ (calcd for C_15_H_18_O_3_Na, 269.1154).

### Computational methods

Conformers of **1**–**3** were generated using the GMMX add-on module of GaussView 6 with an energy window of 10 kcal/mol. Optimization of suggested conformers followed by TDDFT calculations were performed using Gaussian 16 with various combinations of functionals (B3LYP, CAM-B3LYP, APFD, ωB97X-D) and basis sets [6-311+G(d,p), 6-31+G(d,p)] with the CPCM solvent model. ECD spectra were generated by the SpecDis program using a Gaussian band shape with 0.3–0.35 eV [24, 25]. The overall CD spectra of the obtained conformers were Boltzmann weighted at 298 K after UV correction.

## Electronic supplementary material

Below is the link to the electronic supplementary material.

Supplementary file1 (PDF 7337 kb)
