# Correction to: Sesquiterpenes with new carbon skeletons from the basidiomycete *Phlebia tremellosa*

**DOI:** 10.1007/s11418-020-01403-y

**Published:** 2020-04-10

**Authors:** Ken-ichi Nakashima, Junko Tomida, Takao Hirai, Yoshiaki Kawamura, Makoto Inoue

**Affiliations:** 1 Laboratory of Medicinal Resources, School of Pharmacy, Aichi Gakuin University, 1-100 Kusumoto-cho, Chikusa-ku, Nagoya, Aichi 464-8650 Japan; 2 Department of Microbiology, School of Pharmacy, Aichi Gakuin University, 1-100 Kusumoto-cho, Chikusa-ku, Nagoya, Aichi 464-8650 Japan

## Correction to: Journal of Natural Medicines (2019) 73:480–486 https://doi.org/10.1007/s11418-019-01286-8

The article Sesquiterpenes with new carbon skeletons from the basidiomycete *Phlebia tremellosa*, written by Ken ichi Nakashima, Junko Tomida, Takao Hirai, Yoshiaki Kawamura and Makoto Inoue was originally published Online First without Open Access. After publication in volume 73 issue 3, page 480–486 the author decided to opt for Open Choice and to make the article an Open Access publication. Therefore, the copyright of the article has been changed to © The Author(s) 2020 and the article is forthwith distributed under the terms of the Creative Commons Attribution 4.0 International License (https://creativecommons.org/licenses/by/4.0/), which permits use, duplication, adaptation, distribution and reproduction in any medium or format, as long as you give appropriate credit to the original author(s) and the source, provide a link to the Creative Commons license, and indicate if changes were made.

The original article was updated.

