# Supplementary material for: Sesquiterpenes with new carbon skeletons from the basidiomycete Phlebia tremellosa
Source: J Nat Med. 2019 Feb 19;73(3):480–6. doi: 10.1007/s11418-019-01286-8 (PMC7188727; doi:10.1007/s11418-019-01286-8)
Supplement: Supplementary file 1 — Supplementary file1 (PDF 7337 kb) [file 11418_2019_1286_MOESM1_ESM.pdf]

Supplementary data

Sesquiterpenes with new carbon skeletons from the Basidio-mycete *Phlebia tremellosa*

Ken-ichi Nakashima,*,† Junko Tomida,‡ Takao Hirai,† Yoshiaki Kawamura,‡ and Makoto Inoue†

† Laboratory of Medicinal Resources, School of Pharmacy, Aichi Gakuin University, 1-100 Kusumoto-cho, Chikusa-ku, Nagoya, Aichi 464-8650, Japan

‡ Department of Microbiology, School of Pharmacy, Aichi Gakuin University, 1-100 Kusumoto-cho, Chikusa-ku, Nagoya, Aichi 464-8650, Japan

Content

Figure S1. A phylogenetic tree for ECN-184 and related species based on ITS of rDNA sequences....S2

Figure S2. A phylogenetic tree for ECN-184 and related species based on D1/D2 dmain of 26SrDNA sequences....S3

Figure S3. ^{1}H-NMR spectrum of **1** in $CDCl_3$ S4

Figure S4. ^{13}C-NMR and DEPT135 spectra of **1** in $CDCl_3$ S5

Figure S5. DQF-COSY spectrum of **1** in $CDCl_3$ S6

Figure S6. HMQC spectrum of **1** in $CDCl_3$ S7

Figure S7. HMBC spectrum of **1** in $CDCl_3$ S8

Figure S8. NOESY spectrum of **1** in $CDCl_3$ S9

Figure S9. ^{1}H-NMR spectrum of **2** in $CDCl_3$ S10

Figure S10. ^{13}C-NMR and DEPT135 spectra of **2** in $CDCl_3$ S11

Figure S11. DQF-COSY spectrum of **2** in $CDCl_3$ S12

Figure S12. HMQC spectrum of **2** in $CDCl_3$ S13

Figure S13. HMBC spectrum of **2** in $CDCl_3$ S14

Figure S14. NOESY spectrum of **2** in $CDCl_3$ S15

Figure S15. ^{1}H-NMR spectrum of **3** in $CDCl_3$ S16

Figure S16. ^{13}C-NMR and DEPT135 spectra of **3** in $CDCl_3$ S17

Figure S17. DQF-COSY spectrum of **3** in $CDCl_3$ S18

Figure S18. HMQC spectrum of **3** in $CDCl_3$ S19

Figure S19. HMBC spectrum of **3** in $CDCl_3$ S20

Figure S20. NOESY spectrum of **3** in $CDCl_3$ S21

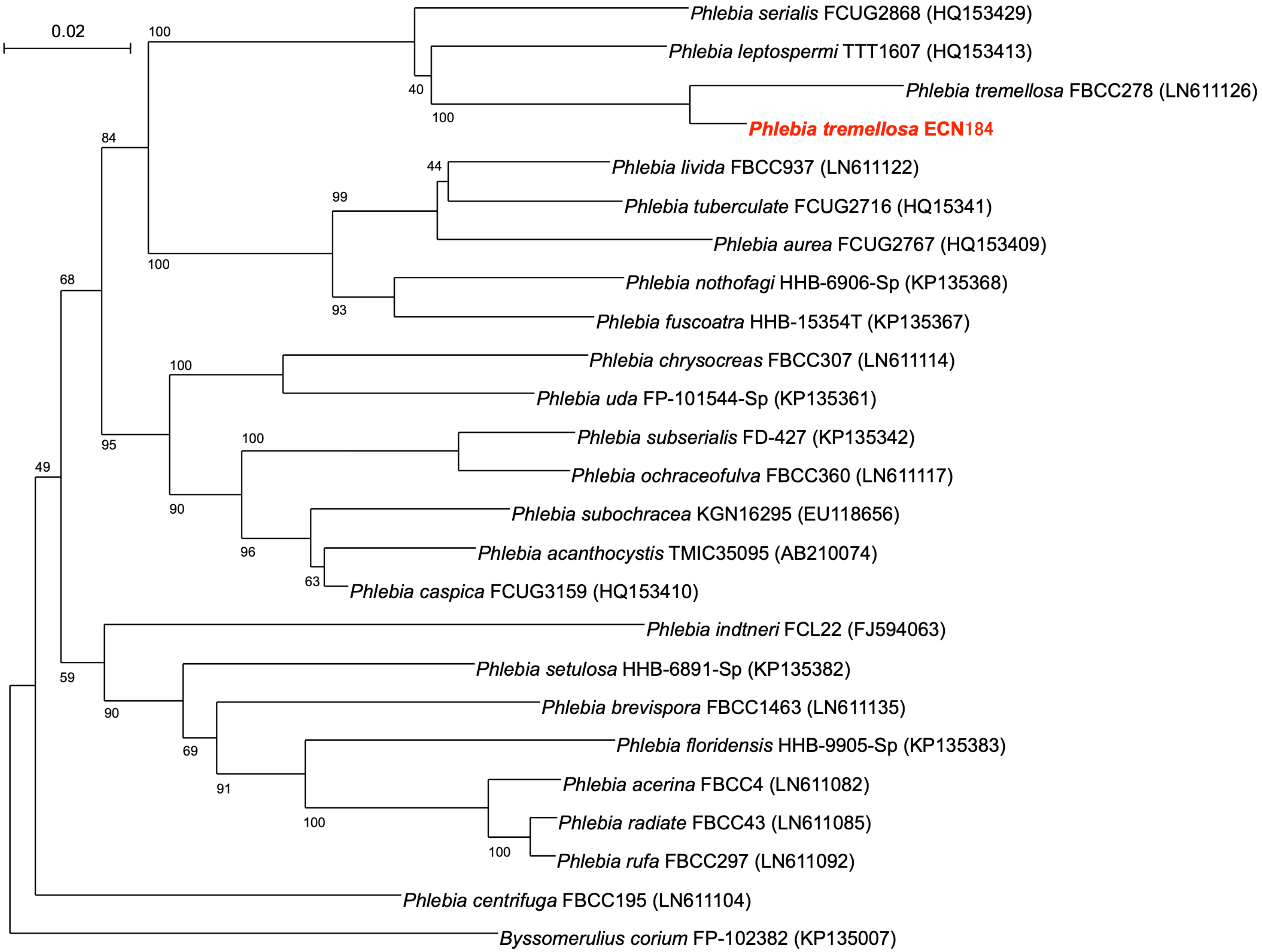

Figure S1. A phylogenetic tree for ECN-184 and related species based on ITS of rDNA sequences

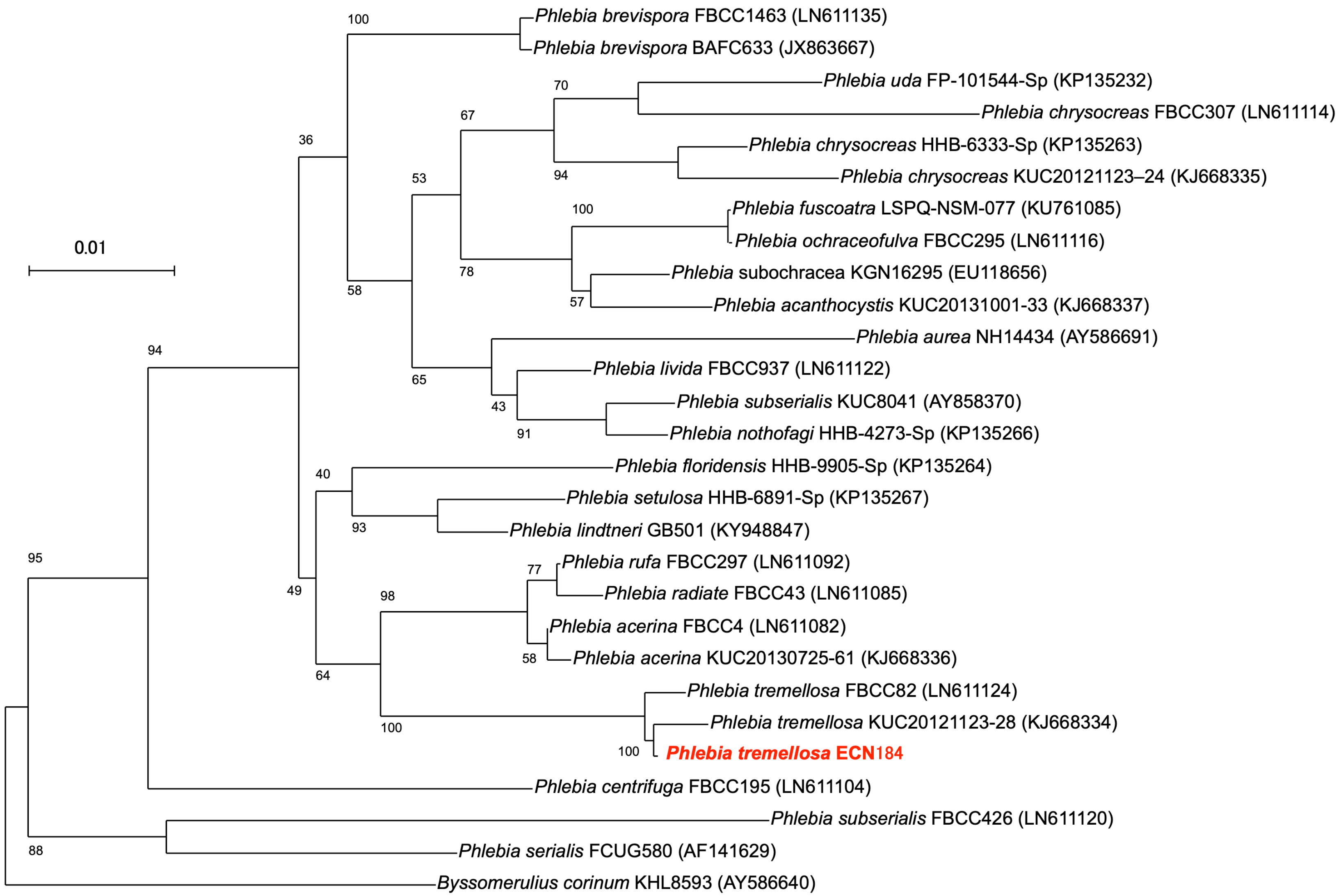

Figure S2. A phylogenetic tree for ECN-184 and related species based on D1/D2 dmain of 26SrDNA sequences

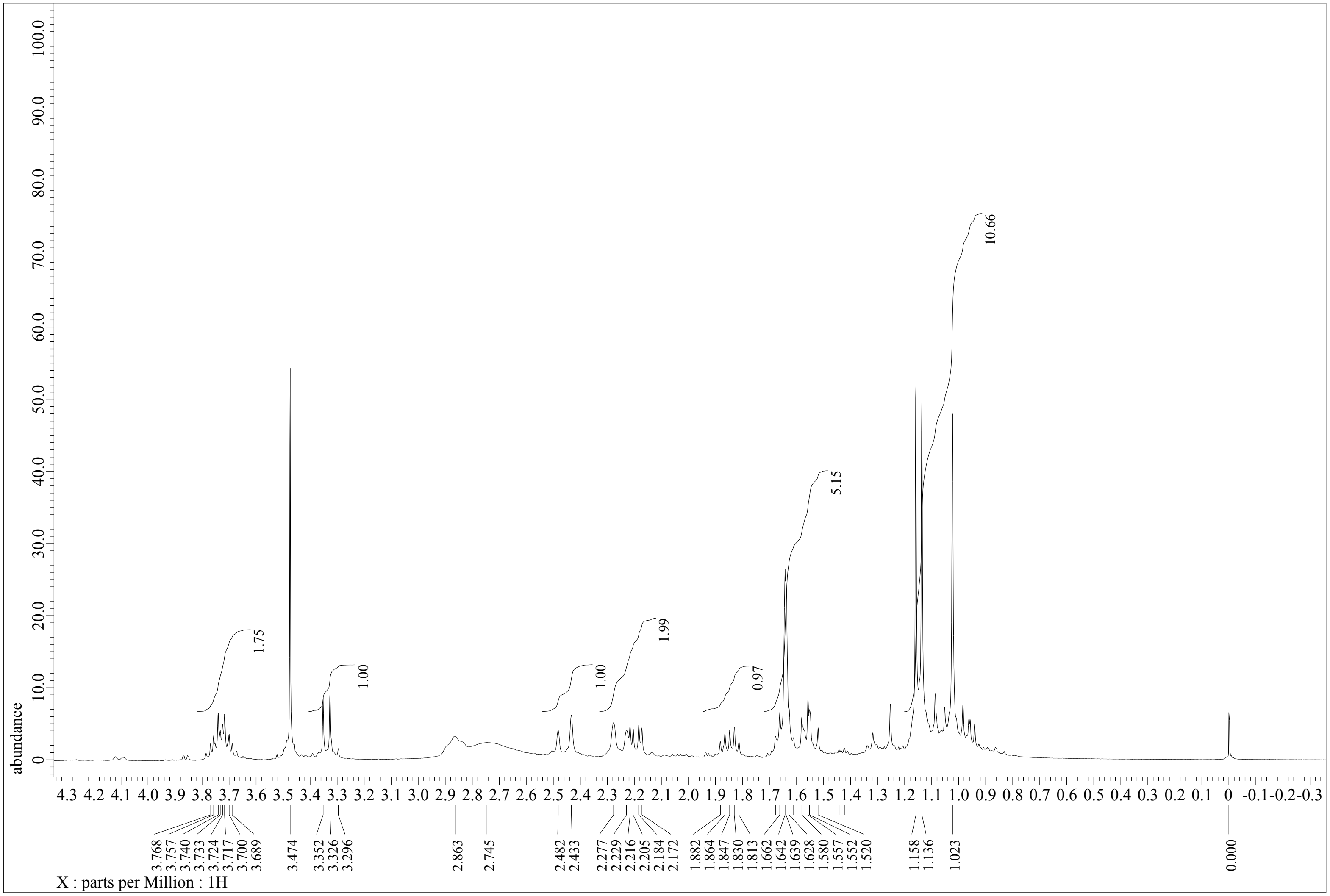

Figure S3. ^{1}H-NMR spectrum of **1** in $CDCl_3$

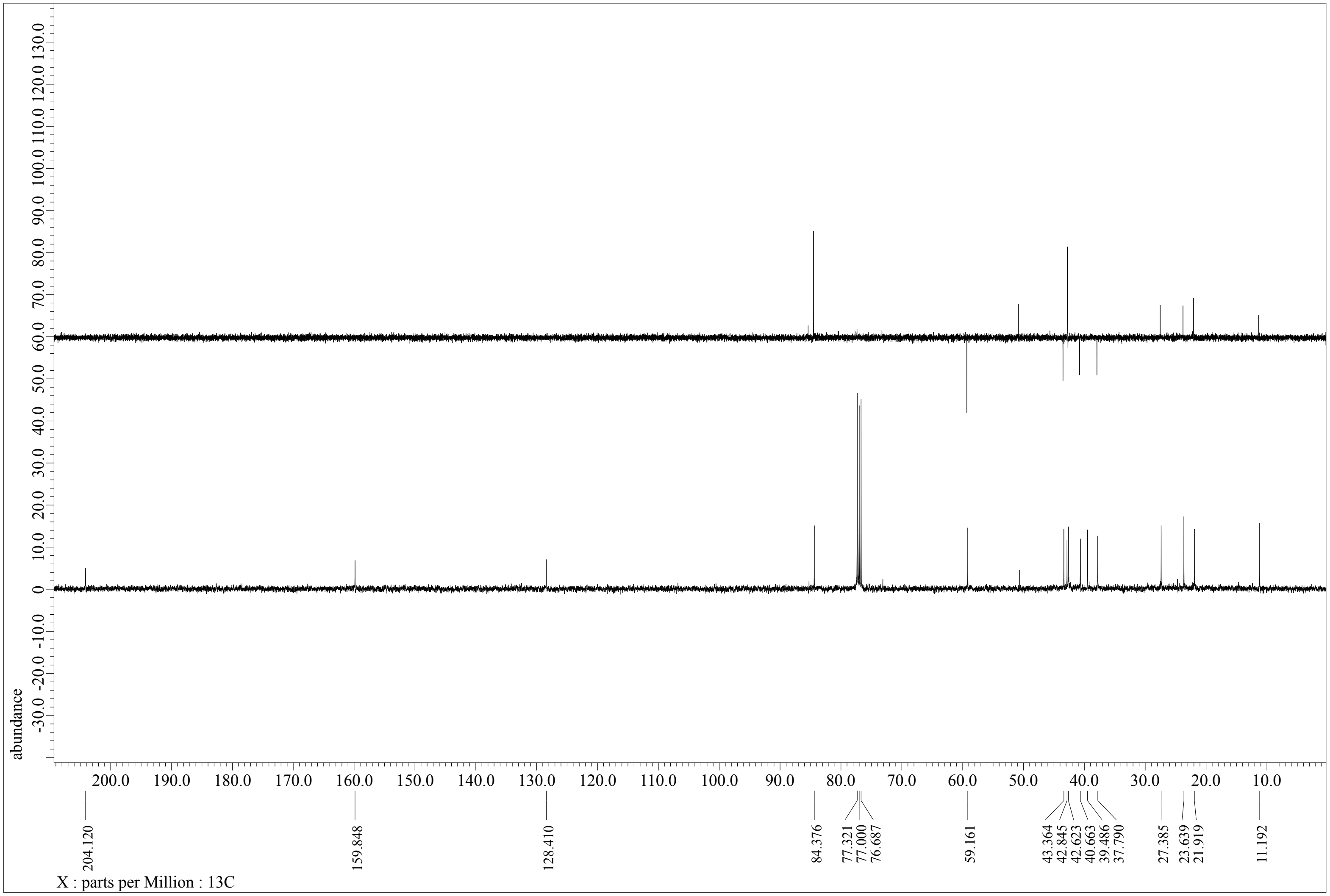

Figure S4. ^{13}C-NMR and DEPT135 spectra of **1** in $CDCl_3$

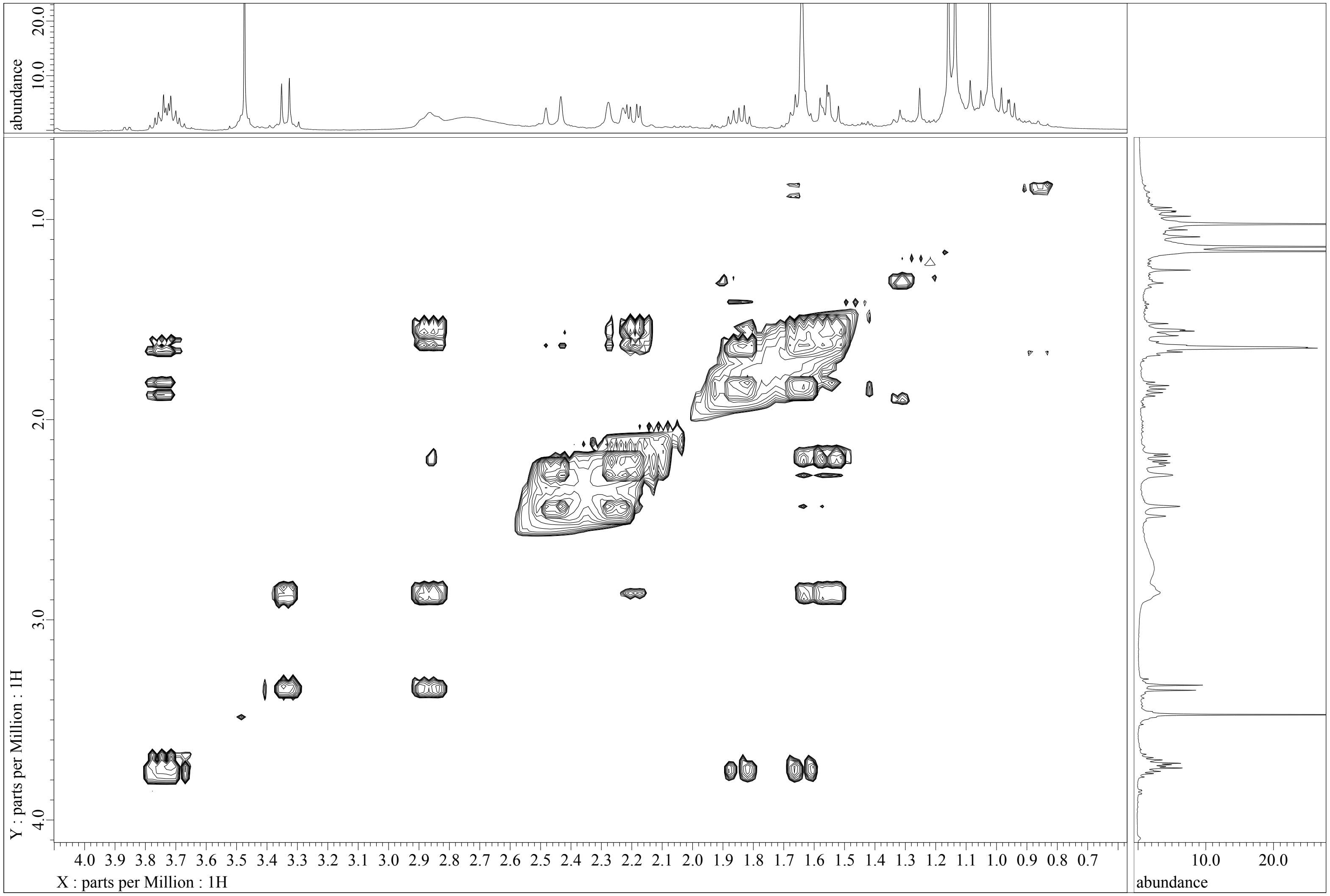

Figure S5. DQF-COSY spectrum of **1** in $CDCl_3$

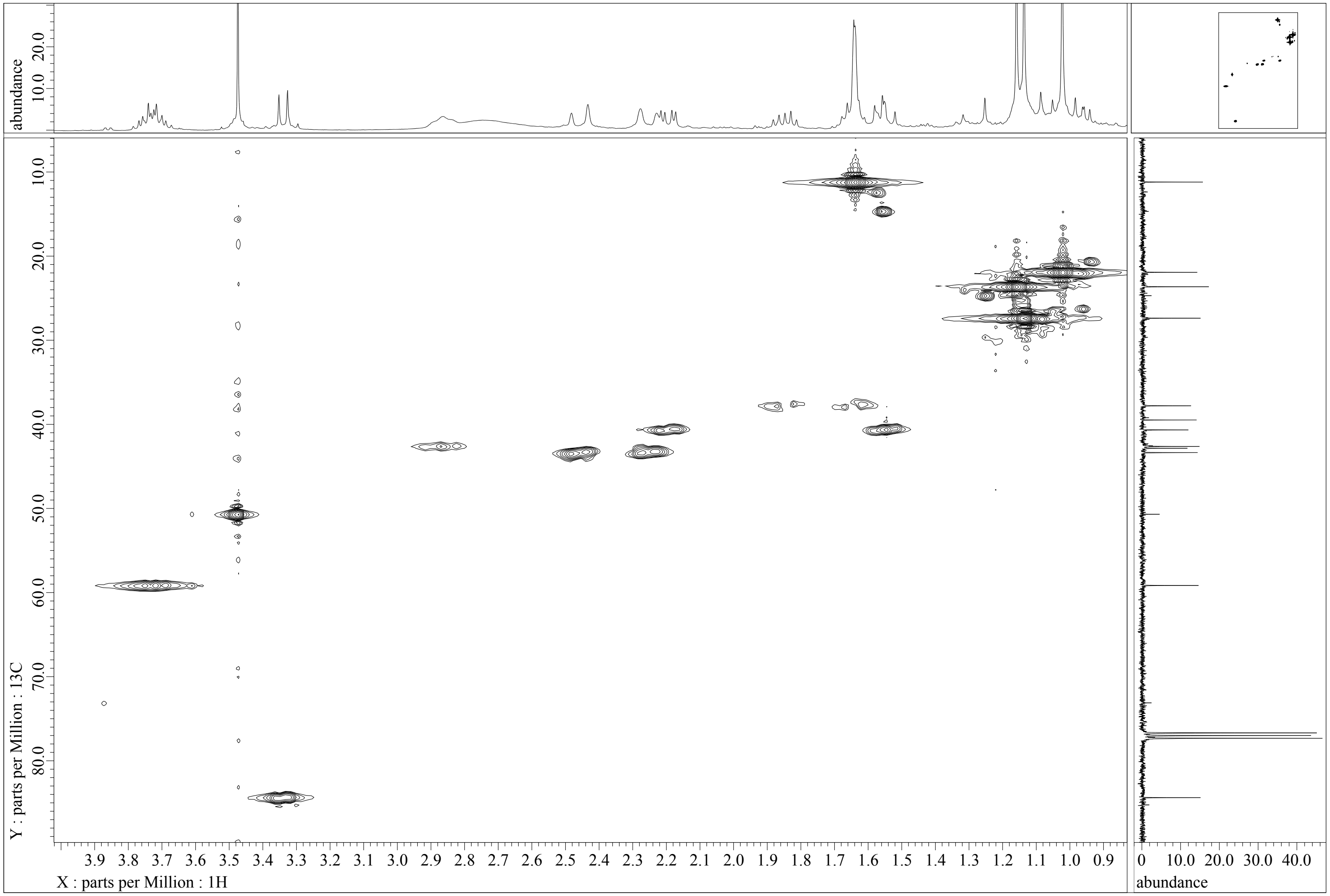

Figure S6. HMQC spectrum of **1** in $CDCl_3$

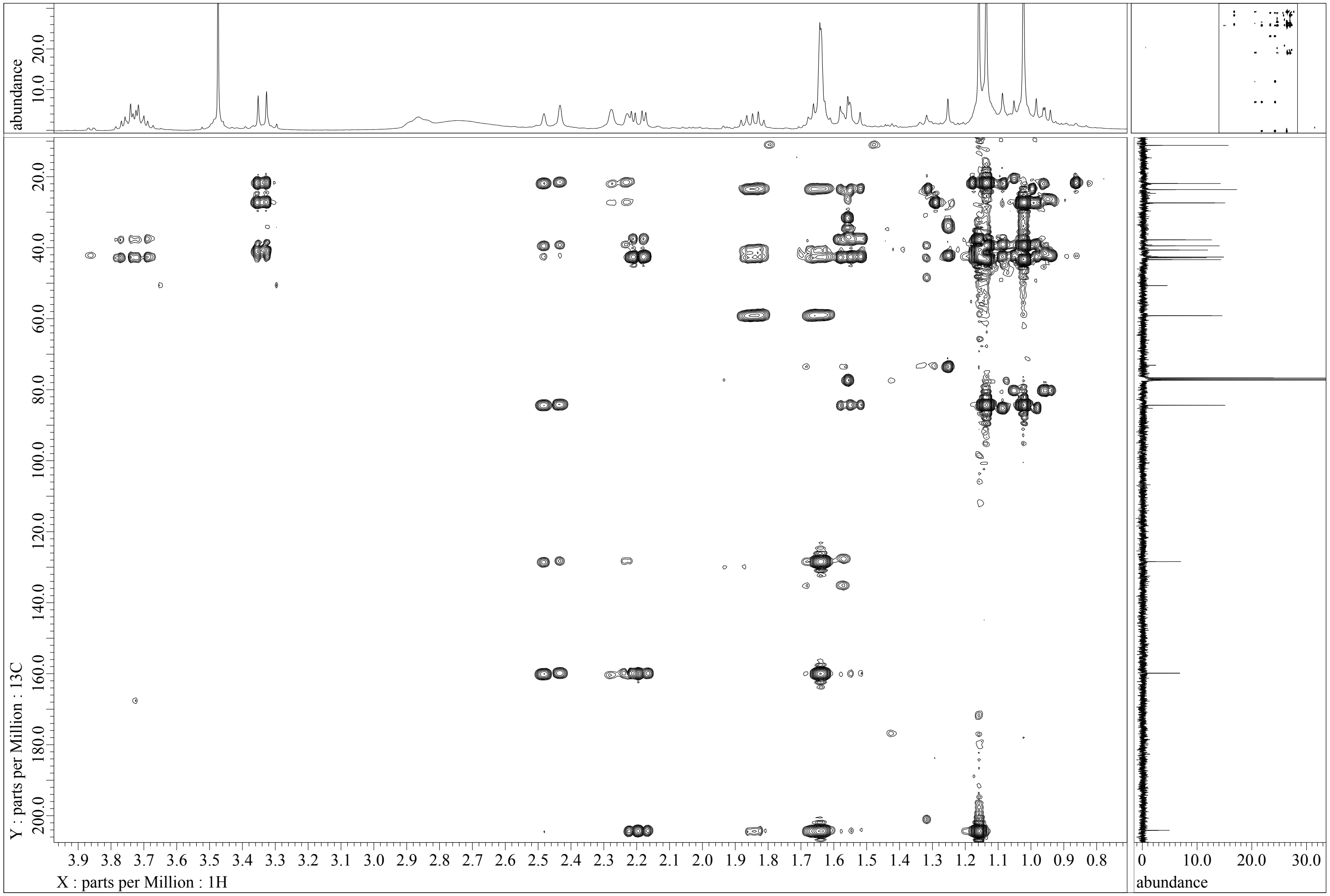

Figure S7. HMBC spectrum of **1** in $CDCl_3$

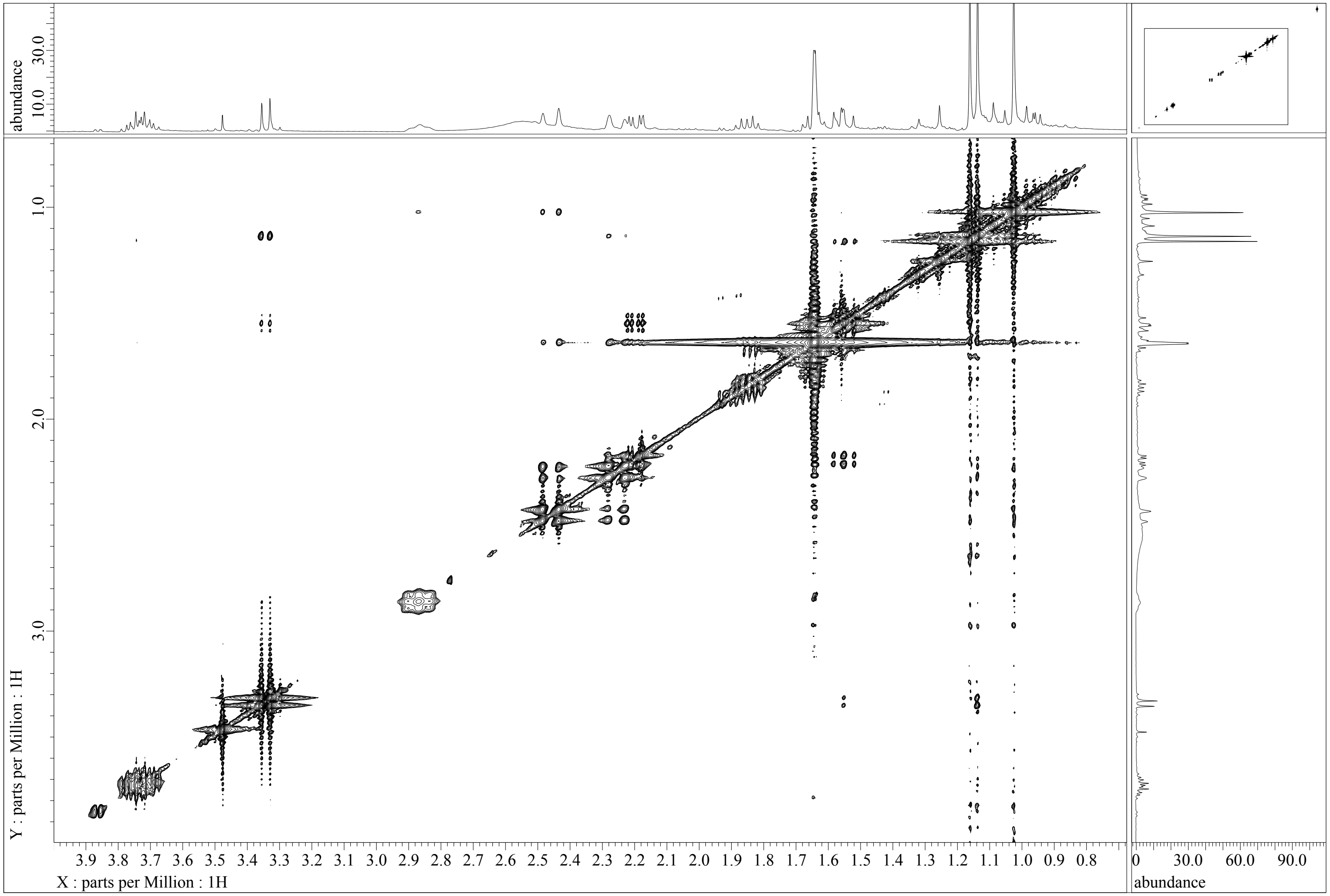

Figure S8. NOESY spectrum of **1** in $CDCl_3$

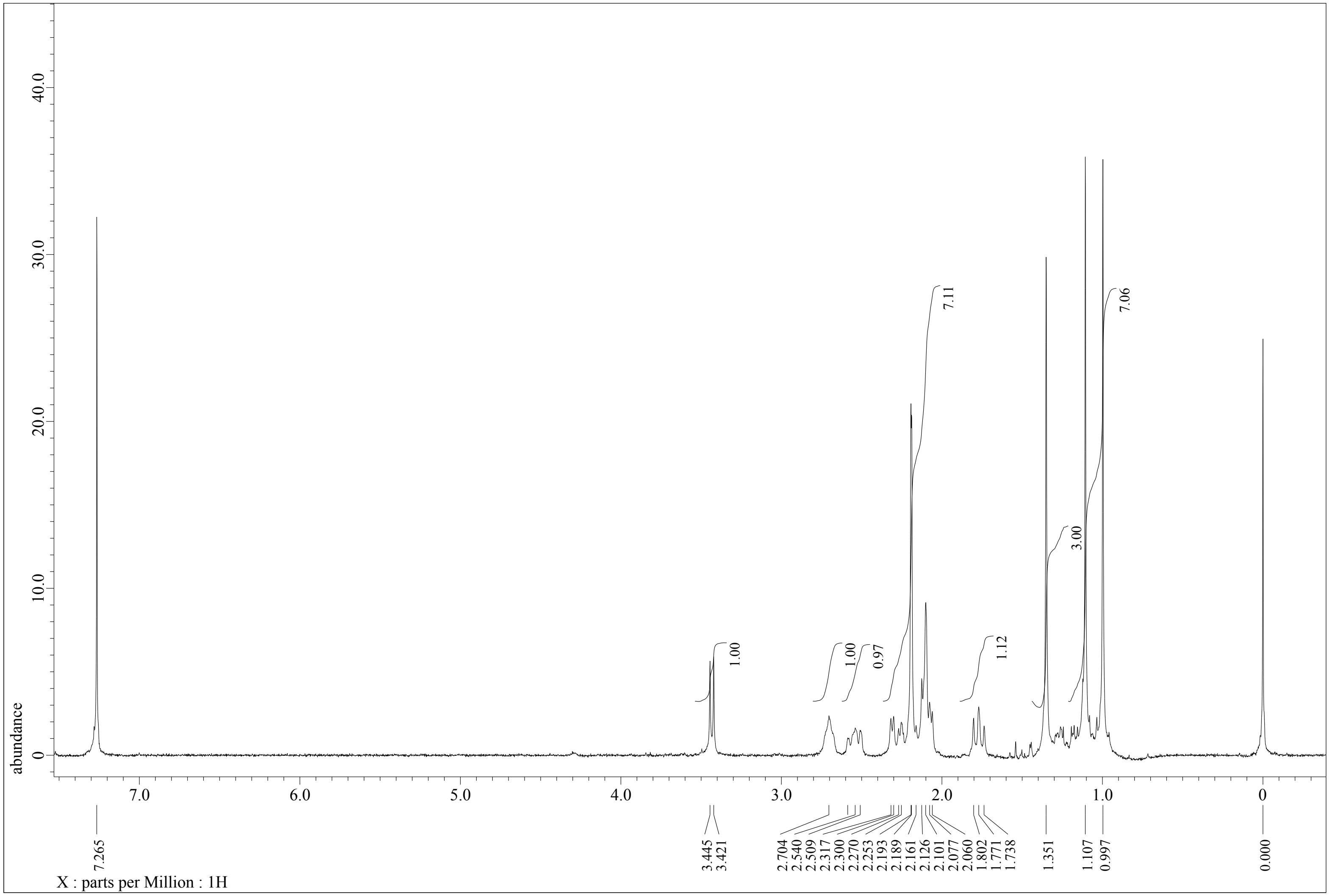

Figure S9. ^{1}H-NMR spectrum of **2** in $CDCl_3$

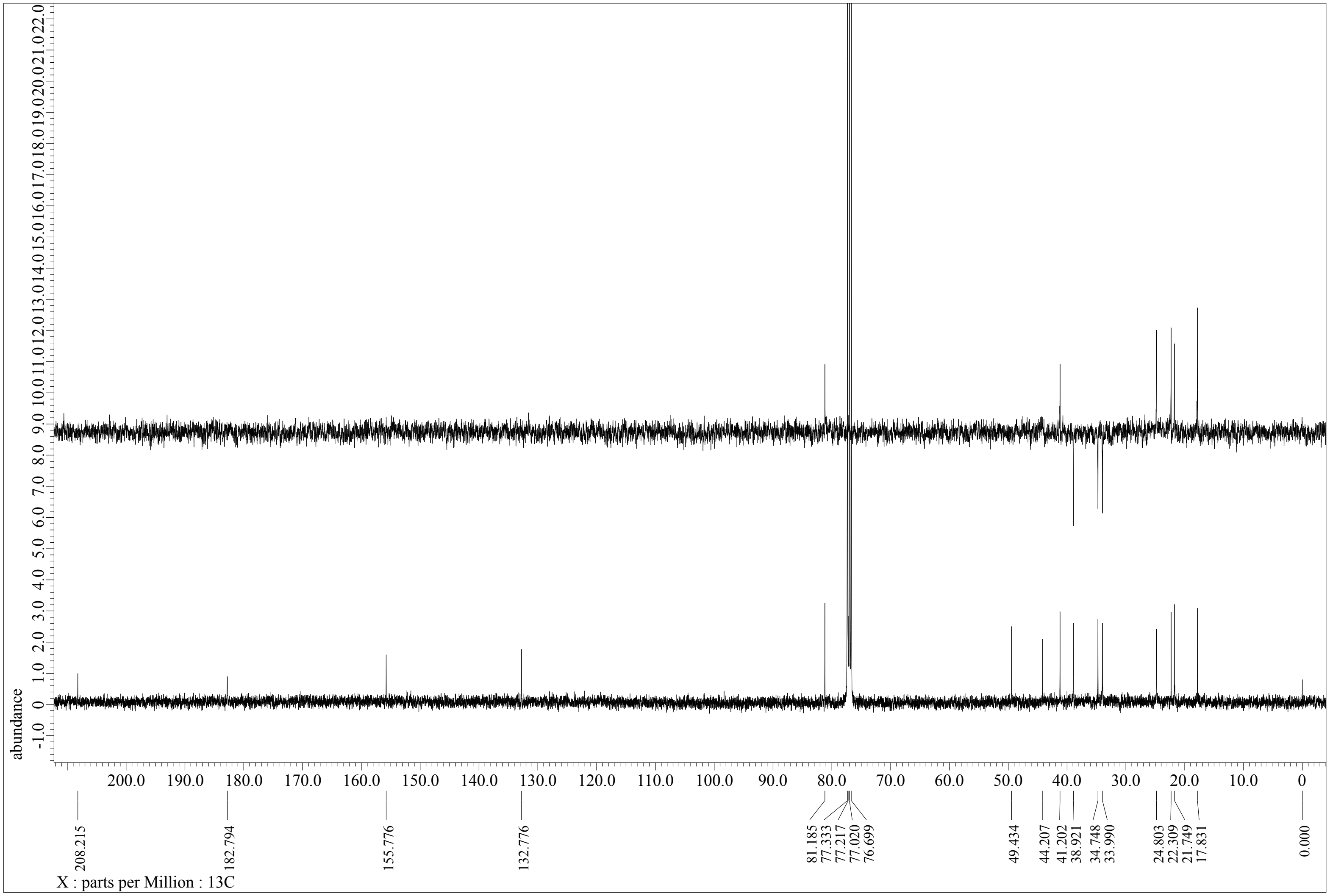

Figure S10. ^{13}C-NMR and DEPT135 spectra of **2** in $CDCl_3$

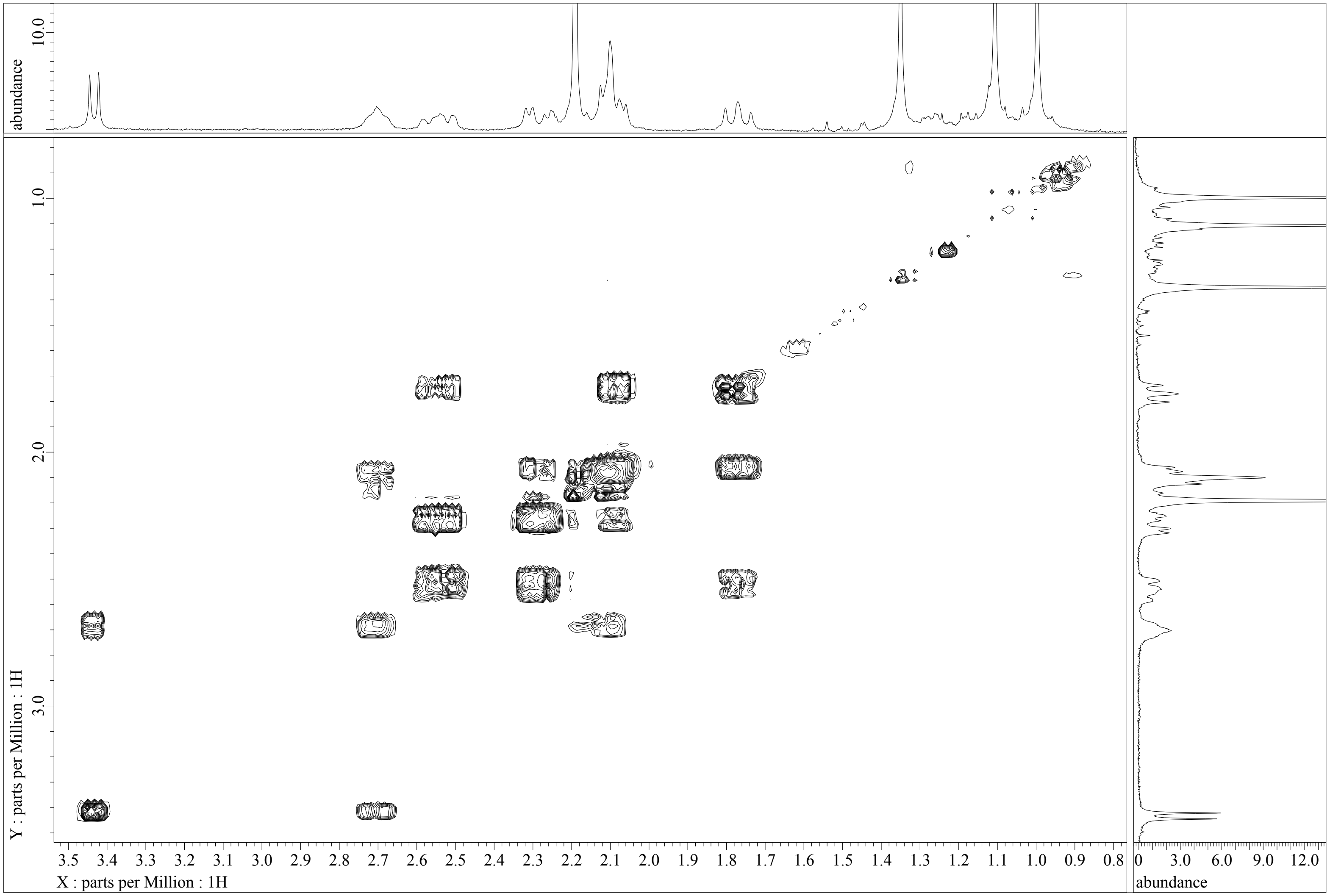

Figure S11. DQF-COSY spectrum of **2** in $CDCl_3$

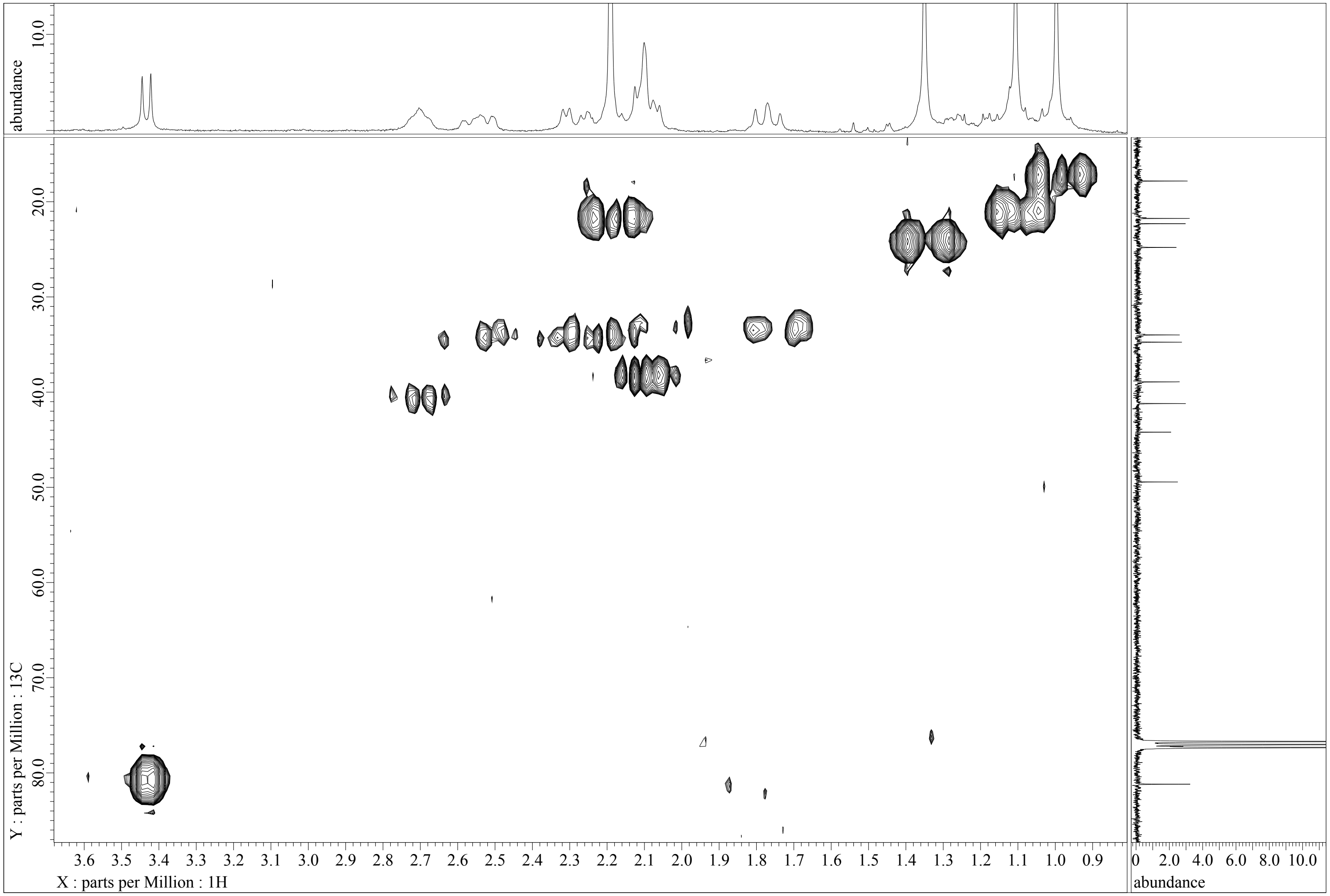

Figure S12. HMQC spectrum of **2** in $CDCl_3$

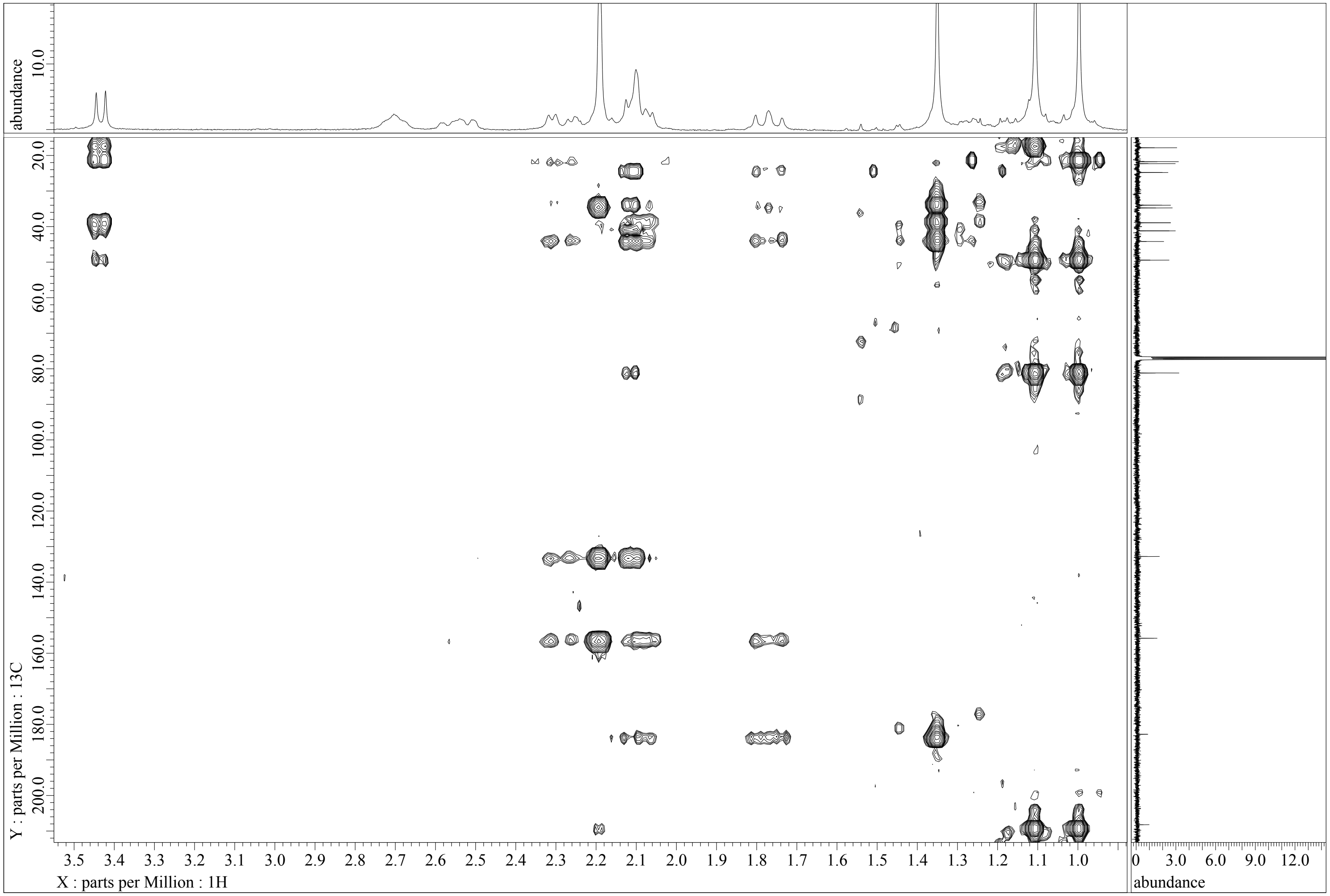

Figure S13. HMBC spectrum of **2** in $CDCl_3$

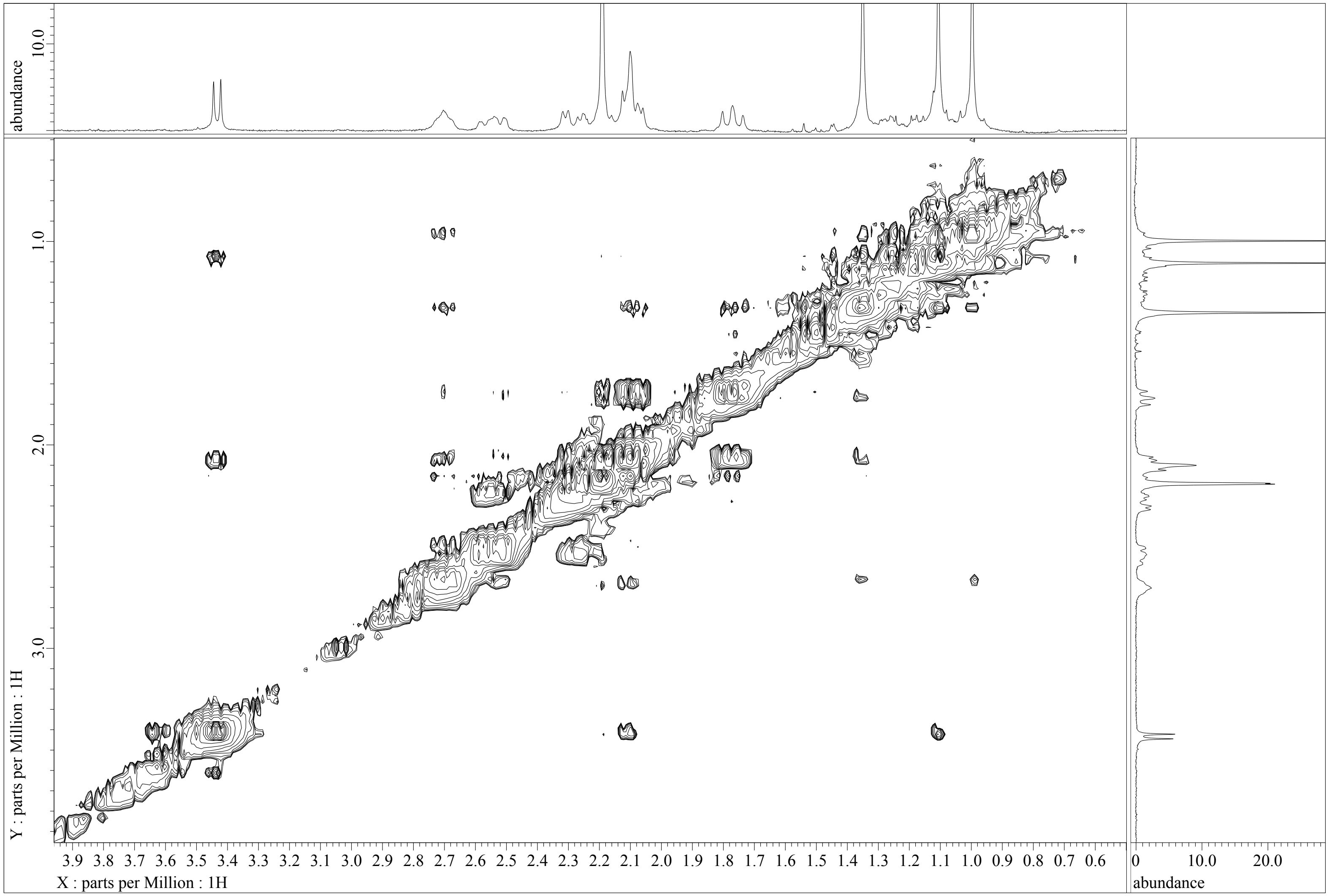

Figure S14. NOESY spectrum of **2** in $CDCl_3$

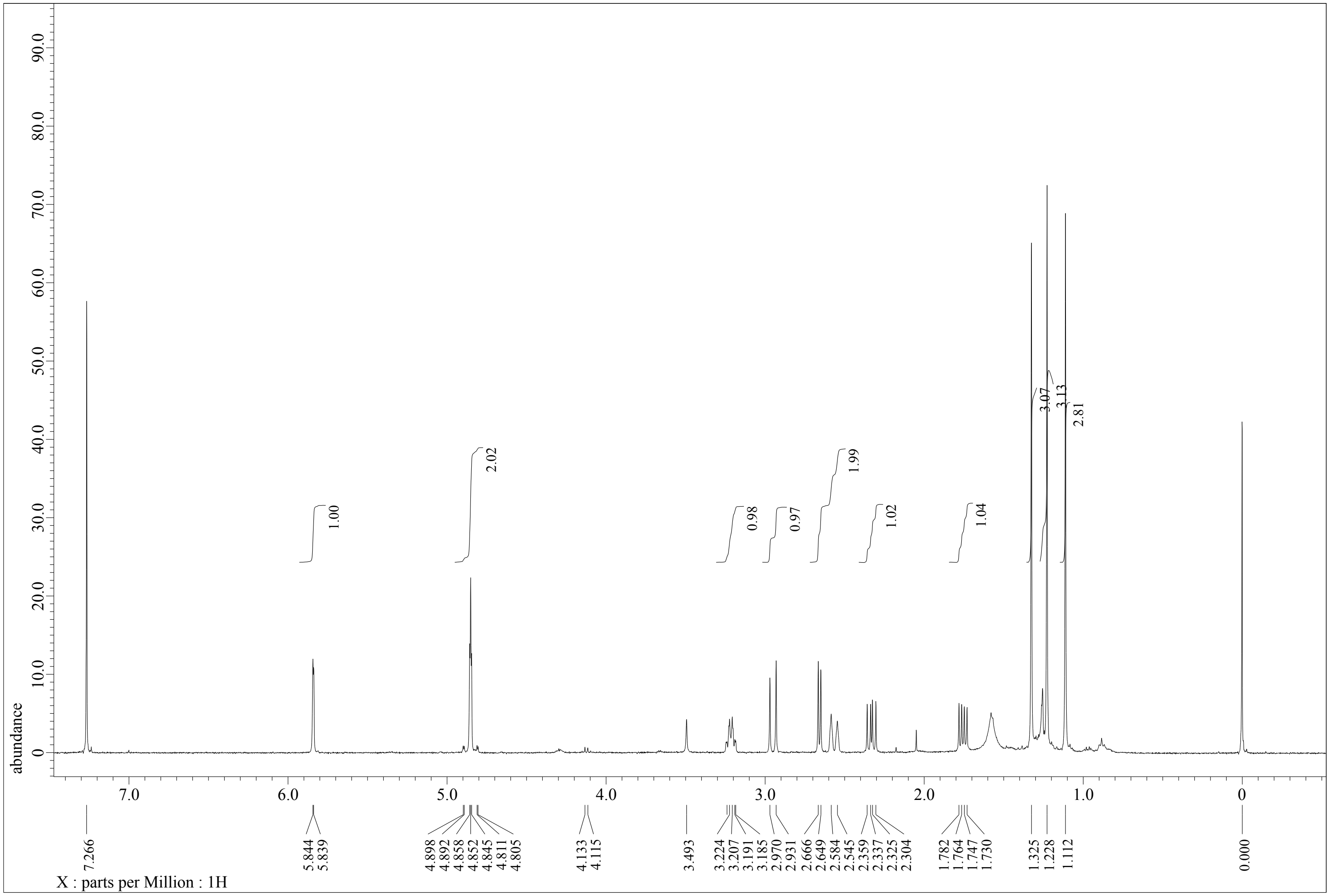

Figure S15. ^{1}H-NMR spectrum of **3** in $CDCl_3$

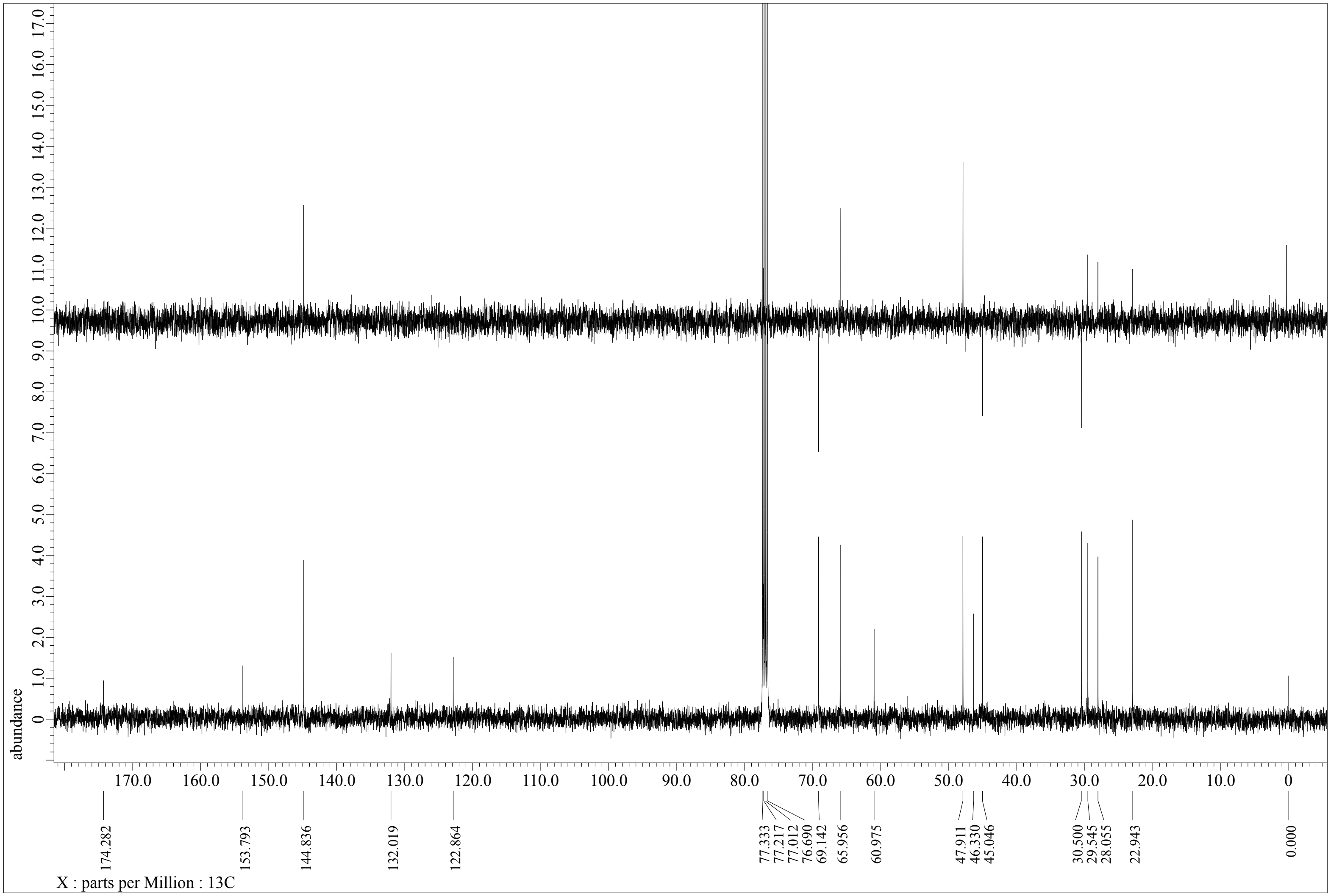

Figure S16. ^{13}C-NMR and DEPT135 spectra of **3** in $CDCl_3$

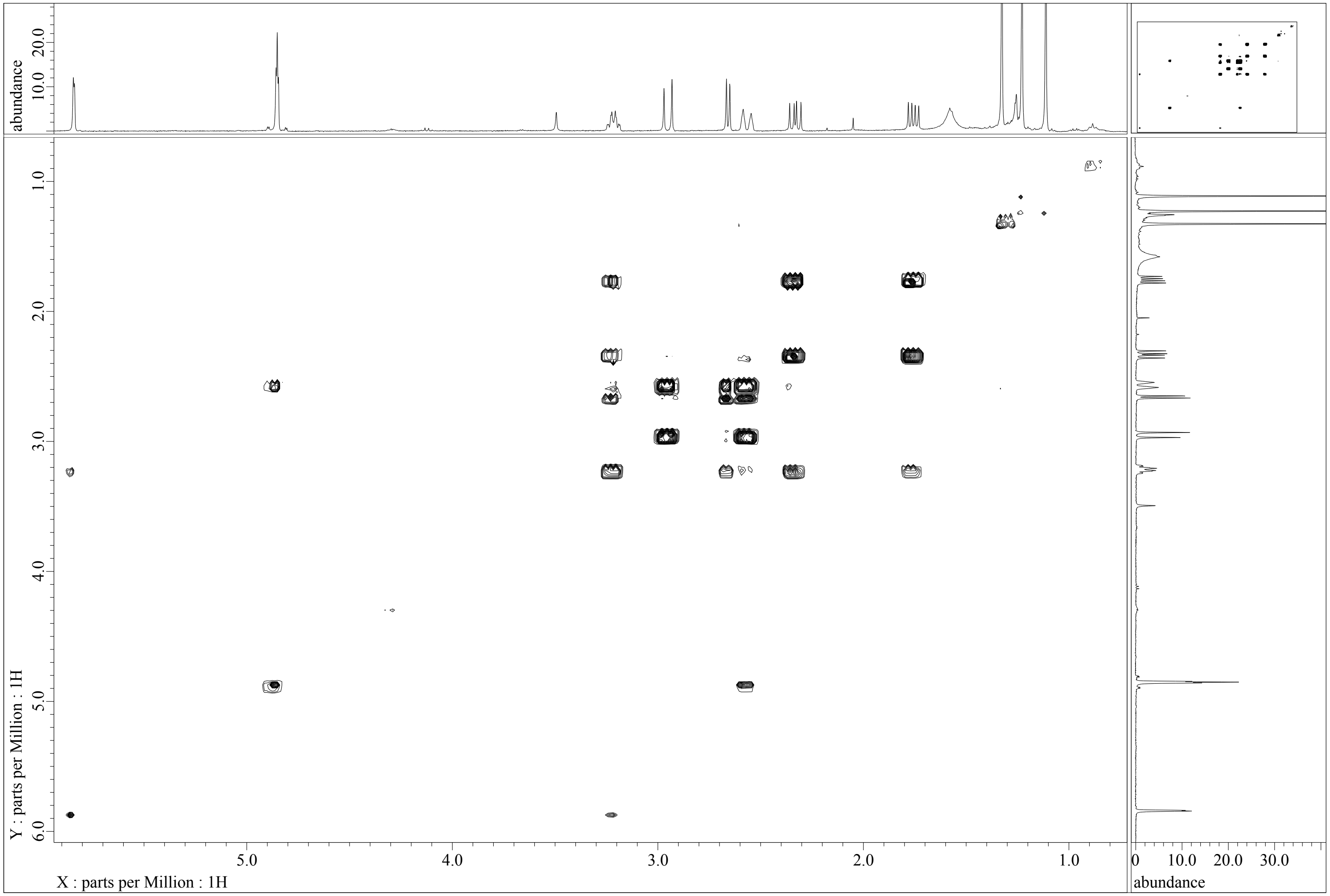

Figure S17. DQF-COSY spectrum of **3** in $CDCl_3$

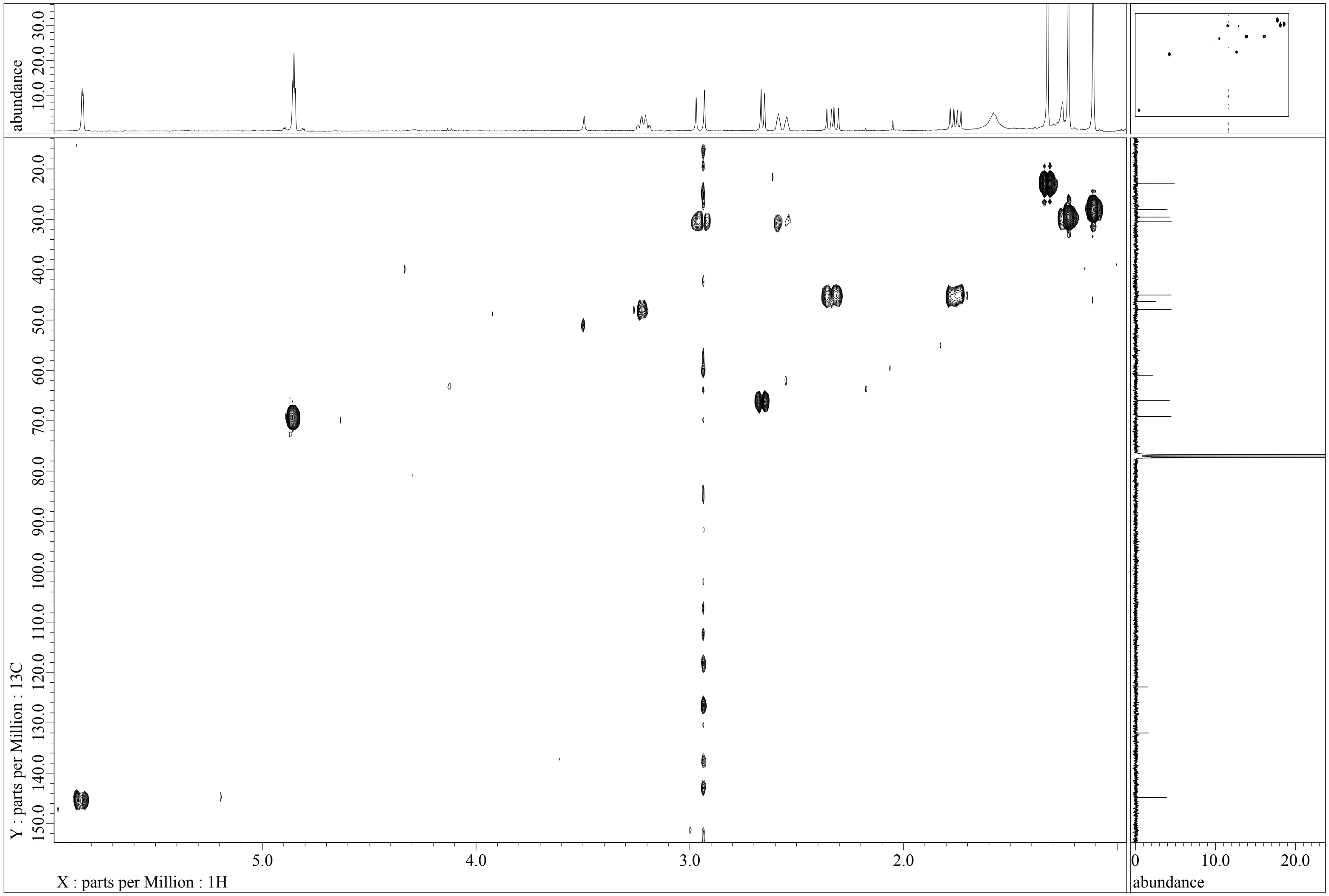

Figure S18. HMQC spectrum of **3** in $CDCl_3$

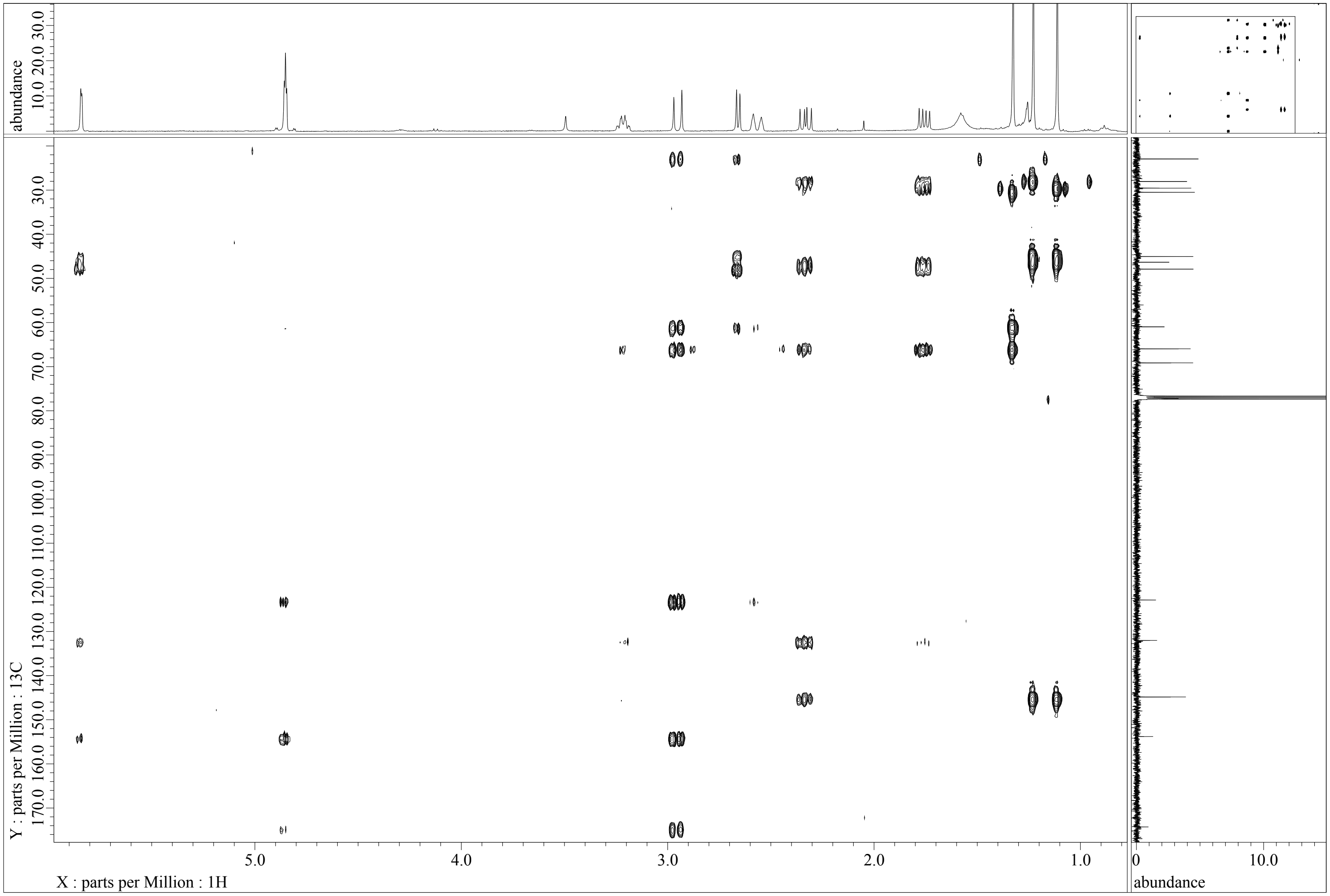

Figure S19. HMBC spectrum of **3** in $CDCl_3$

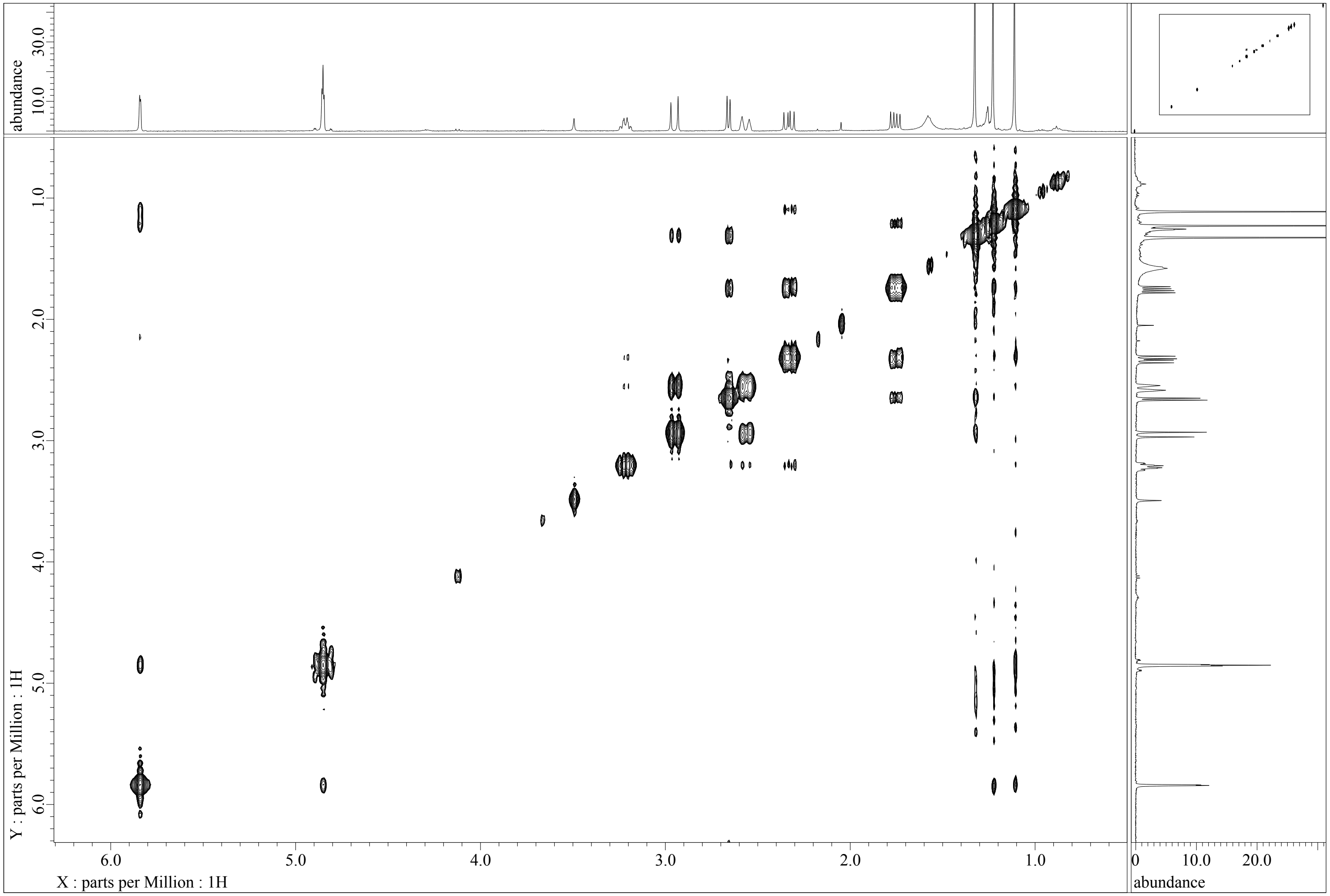

Figure S20. NOESY spectrum of **3** in $CDCl_3$
